# In Vitro Transcription–Translation in an Artificial Biomolecular Condensate

**DOI:** 10.1021/acssynbio.3c00069

**Published:** 2023-06-21

**Authors:** Ludo L. J. Schoenmakers, N. Amy Yewdall, Tiemei Lu, Alain A. M. André, Frank. H.T. Nelissen, Evan Spruijt, Wilhelm T. S. Huck

**Affiliations:** Institute for Molecules and Materials, Radboud University, 6525 AJ Nijmegen, The Netherlands

**Keywords:** biomolecular condensation, coacervation, in vitro transcription translation (IVTT), liquid−liquid phase separation (LLPS), synthetic cell

## Abstract

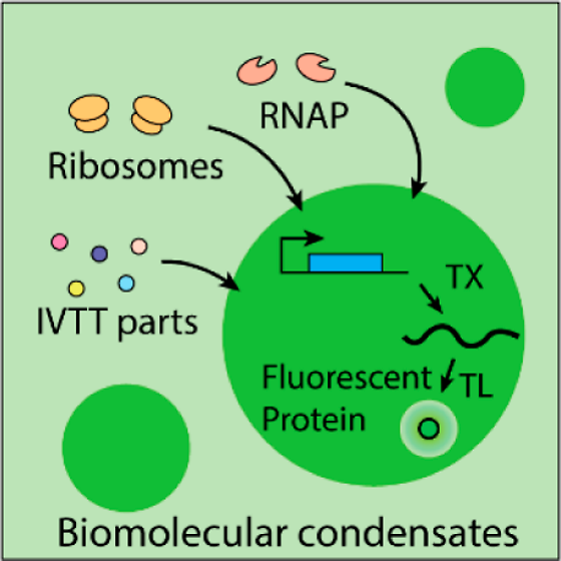

Biomolecular condensates are a promising platform for synthetic cell formation and constitute a potential missing link between the chemical and cellular stage of the origins of life. However, it has proven challenging to integrate complex reaction networks into biomolecular condensates, such as a cell-free in vitro transcription–translation (IVTT) system. Integrating IVTT into biomolecular condensates successfully is one precondition for condensation-based synthetic cell formation. Moreover, it would provide a proof of concept that biomolecular condensates are in principle compatible with the central dogma, one of the hallmarks of cellular life. Here, we have systemically investigated the compatibility of eight different (bio)molecular condensates with IVTT incorporation. Of these eight candidates, we have found that a green fluorescent protein-labeled, intrinsically disordered cationic protein (GFP-K_72_) and single-stranded DNA (ssDNA) can form biomolecular condensates that are compatible with up to μM fluorescent protein expression. This shows that biomolecular condensates can indeed integrate complex reaction networks, confirming their use as synthetic cell platforms and hinting at a possible role in the origin of life.

## 1.

Compartmentalization through phase separation is a universal organizing principle in living cells, from mammalian and plant cells to bacteria.^1−3^ Phase-separated droplets play key roles in biochemical processes, ranging from ribosome biogenesis and RNA processing to signaling. In order to create synthetic biological systems with a complexity matching living cells, an important challenge is to create programmable compartments containing complex biochemical networks,^4^ such as a full in vitro transcription–translation (IVTT) system.^5,6^ Liquid–liquid phase separation (LLPS) is a promising strategy to create droplet-based compartments with a crowded, cytomimetic interior that allow the free exchange of nutrients and waste products of biochemical networks.^7^ Such phase-separated droplets could also provide a greater understanding of early protocells at the origins of life,^8−11^ and serve as a platform for synthetic systems that capture essential biochemical and biophysical hallmarks of living systems.^12−14^

Previous attempts to achieve transcription and translation inside phase-separated droplets yielded varying degrees of success, while illustrating the challenges faced by such platforms (Table S1).^15−19^ In one of the first examples of protein expression inside a phase-separated droplet, Sokolova et al. have shown low micromolar levels of green fluorescent protein (GFP) expression inside a polyethylene glycol (PEG)-rich crowded droplet inside a water-in-oil emulsion.^15^ However, the authors did not determine whether GFP expression took place exclusively inside the dense phase, or that it also took place in the dilute phase followed by partitioning into the dense phase. Another example of protein expression inside a complex coacervate droplet was reported by Tang and co-authors.^16^ IVTT expressing mCherry was added to a carboxymethyl-dextran (CM-dextran)/polylysine (pLys) system. When this mixture was incubated, mCherry was expressed, albeit at low nanomolar levels and only at low temperatures to avoid protein aggregation. Furthermore, for this bottom-up system, it remained unclear whether mCherry expression could also be localized by sequestration inside phase-separated droplet compartments in equilibrium with a surrounding solution, as information about the distribution of key components of the IVTT machinery was lacking, and the differences between IVTT-loaded samples and background fluorescence was small. Recently, Xu and coauthors have presented a partially top-down approach, where *Escherichia coli* and *Pseudomonas aeruginosa* cells were encapsulated and lyzed inside poly(diallyldimethylammonium chloride) (PDADMAC)/adenosine triphosphate (ATP) coacervate droplets.^17^ These bacteria-derived protocells were reported to be capable of deletion enhanced green fluorescent protein (deGFP) expression.^20^ However, fluorescent protein expression was in the low nanomolar range, and the surrounding bacteria-derived membrane makes it difficult for the produced protein to be used in downstream pathways in other compartments.

Here, we report a bottom-up biomolecular condensate system capable of robust deGFP expression inside droplets. We began with eight candidate systems (see Table S2 for an overview): a fusion of GFP with an elastin-like polypeptide (ELP)^21^ here called GFP-K_72_/single-stranded DNA (ssDNA) or torula yeast total RNA (tyRNA),^22,23^ spermine/polyadenine (polyA) or polyuracil (polyU),^24^ nucleophosmin-1 (NPM1)/total *E. coli* ribosomal RNA (rRNA),^25,26^ ATP/polylysine (pLys),^27,28^ protamine sulfate (prot. sulf.)/citrate,^29^ and poly(diallyldimethylammonium) chloride (PDADMAC)/polyacrylic acid (PAA).^30^ We focused on systems that could in theory be programmed into an IVTT system due to their biomolecular nature. Additionally, these systems cover different possible combinations of long versus short length, and high versus low charge density polycations and polyanions. We hypothesized that these are important parameters that affect droplet stability and IVTT uptake into the droplets. The only fully synthetic system, PDADMAC/PAA, has been added due to the previous use of PDADMAC in similar work.^16,17,30^

**Figure 1:**
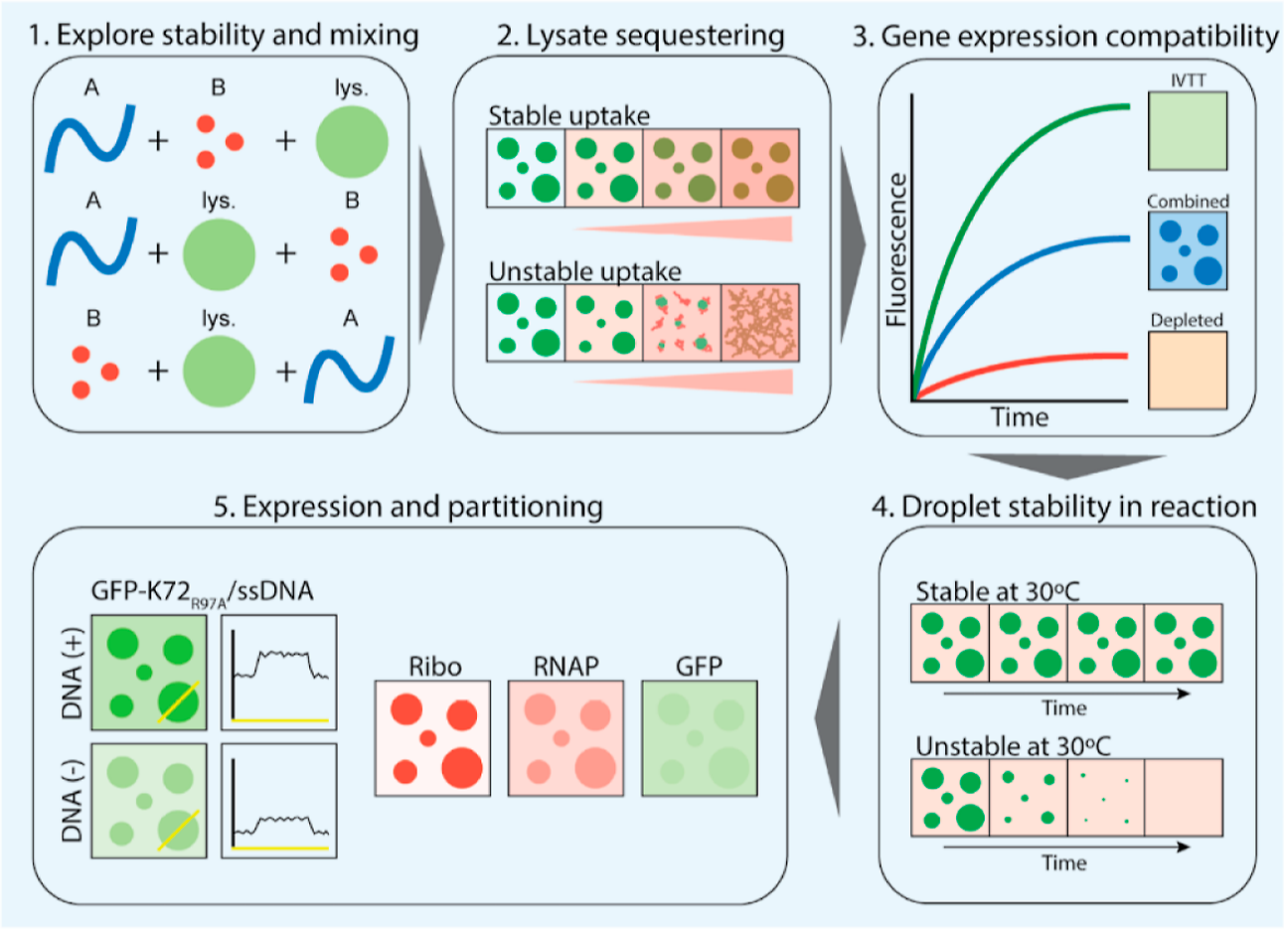
Approach. The five steps used to determine which candidate system is compatible with in-droplet IVTT: (1) exploration of condensate formation, stability, and the role of the order of mixing; (2) uptake of cell lysate into condensates; (3) determine the compatibility of condensates with gene expression; (4) establishing the stability of lysate-containing condensate droplets under full reaction conditions; (5) measurement of protein expression in condensates, as well as the partitioning of labeled *E. coli* RNAP and ribosomes.

For each of these systems, we systemically determined its compatibility with in-droplet expression. Our approach consisted of five steps (Figure 1). First, we determined the mixing order of the charged components of each system with a dilute, labeled bacterial cell lysate. Second, we investigated the effect of increased lysate concentrations, as well as an IVTT reaction mixture, on droplet morphology and stability. Third, we explored the compatibility of the most promising systems with expression by testing the capacity to sequester the necessary IVTT components and express a fluorescent protein (deGFP). Fourth, we determined the droplet stability over time under reaction conditions. Fifth and finally, we determined deGFP expression in a droplet sample using confocal microscopy.

Ultimately, we found that a system consisting of a lysine-rich ELP fused to a non-fluorescent GFP mutant (GFP-K_72_-R97A) in combination with synthetic ssDNA ((ACTG)_11_) was capable of deGFP expression in the μM range. Here, GFP initially served as an expression tag for bacterial overexpression, as K_72_ expression is toxic.^21,31^ A non-fluorescent version was created for compatibility with deGFP expression. Additionally, we determined that *E. coli* RNAP and the *E. coli* 70S ribosome, two key biomolecular components for transcription and translation, partition into GFP-K_72_-R97A/ssDNA droplets, whereas purified enhanced green fluorescent protein (eGFP) does not. Together, these results show that deGFP can be expressed inside GFP-K_72_-R97A/ssDNA droplets using a bottom-up approach.

## 2. Results and Discussion

### 2.1 Exploration of Coacervate Formation

**Figure 2:**
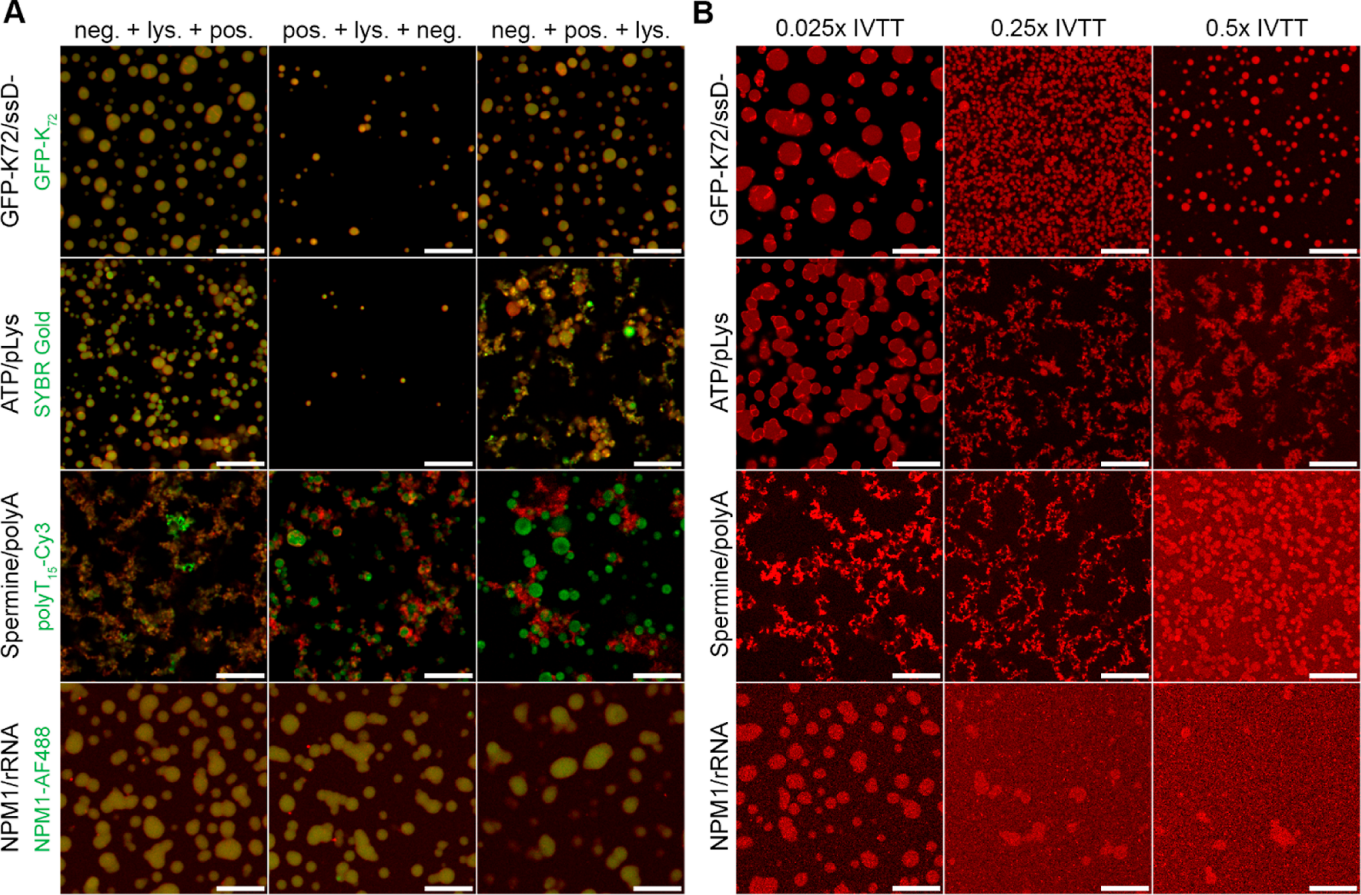
Mixing order and IVTT sequestering in four systems. (A) Partitioning of 0.25 mg/mL lysate labeled with Alexa Fluor 647 (AF647) into condensate droplets. Overlay of labeled coacervates (green channel) and labeled lysate (red channel). GFP-K_72_/ssDNA green channel = GFP-K_72_. ATP/pLys green channel = SYBR Gold (also stains ATP as shown previously^57^). Spermine/polyA green channel = polyT_15_-Cy3 (at 554 nm). NPM1/rRNA green channel = 9:1 NPM1:NPM1-AF488. Final compositions: GFP-K_72_/ssDNA: 12 μM GFP-K_72_, 0.025 mg/mL ssDNA, 2 mM Tris-HCl pH 7.4, 5 mM MgCl_2_. ATP/pLys: 5 mM ATP, 5 mM pLys, 10 mM Tris-HCl pH 7.4, 100 mM NaCl, 5 mM MgCl_2_. Spermine/polyA: 10 mM spermine, 1 mg/mL polyA, 10 mM Tris-HCl pH 7.4, 1 mM MgCl_2_. NPM1/rRNA: 20 μM NMP1, 0.2 mg/mL rRNA, 10 mM Tris-HCl pH 7.4, 150 mM NaCl. Three mixing orders: (1) negatively charged component plus lysate, followed by the positively charged components, (2) positively charged component plus lysate, followed by the negatively charged components, and (3) negatively charged component plus positively charged components, followed by lysate. (B) Effect of an increasing concentration of the IVTT reaction mixture on droplet stability and morphology of GFP-K_72_/ssDNA, ATP/pLys, spermine/polyA, and NPM1/rRNA droplets. Structures visualized by looking at AF647-labeled lysate, showing a difference in lysate partitioning into the droplets. For the precise IVTT composition, see Table S5. All scale bars represent 20 μm.

We first explored the effect of the mixing order of the positively and negatively charged components of each system together with a low concentration (0.25 mg/mL total protein) of an *E. coli* cell lysate on droplet formation (Figures 2A and S3). This cell lysate was produced and labeled in-house with Alexa Fluor 647 (see Materials and MethodsSection 4.10). Here, Figure 2 shows the four systems which ultimately looked most promising throughout our five-step approach (Figure 1, Table S8). For the first step, the positively charged component, negatively charged component, and labeled lysate were mixed in the three possible orders. Most systems showed droplet formation and labeled lysate uptake for at least one mixing order. In general, mixing the negatively charged component with lysate before the addition of the positively charged component gave more stable and numerous droplets than first mixing the positively charged component with lysate. This can be explained in terms of the net negative surface charge of the proteins and polynucleotides present in the cell lysate,^32,33^ which can sequester the positively charged component, thereby preventing droplets from forming. Out of the eight systems, only spermine/polyA and spermine/polyU showed extensive aggregation, which can be explained by the strong interaction between spermine and the negatively charged biomolecular components of the lysate.

### 2.2 Sequestration of Bacterial Cell Lysate into Coacervates

Based on the ideal mixing orders (Figures 2A and S3), we next explored the effect of an increased lysate concentration on droplet formation (Figure S4). We found that a lysate concentration up to 10 mg/mL total protein led to aggregation for spermine/polyU, spermine/polyA, ATP/pLys, prot. sulf./citrate, and PDADMAC/PAA. However, a full IVTT reaction is made up of both lysate and a high ionic strength feeding buffer. This feeding buffer may heavily influence droplet formation. Thus, we next tested the effect of a standard IVTT reaction mixture on droplet morphology and stability (see Table S5 for an overview of the IVTT compositions used in this study). At this point, we defined a stable system as one which shows a similar droplet number and/or size as compared to the mixing order experiment (Figures 2A and S3).

As the IVTT concentration was increased up to 0.5× of the typical component concentrations,^5^ systems either showed numerous droplets (GFP-K_72_/ssDNA, spermine/polyA), a reduced number of droplets (GFP-K_72_/tyRNA, NPM1/rRNA), or aggregation (ATP/pLys, prot. sulf./citrate, PDADMAC/PAA) (Figures 2B and S6). Aggregate formation was a particular problem for systems with a high charge density, such as ATP/pLys,^27^ protamine sulfate/citrate,^29^ and PDADMAC/PAA.^34^ Interestingly, spermine/polyA was stabilized as the IVTT concentration increased, showing clear partitioning of labeled lysate into the droplets (Figure 2B). Most likely, the high ionic strength of the IVTT mixture allows for the conversion of precipitates to droplets, which has been previously reported in polyelectrolyte droplet systems.^35^ Finally, for NPM1/rRNA, the reduced number of droplets can be explained by the ionic strength of IVTT exceeding the upper limit for droplet formation.^25^

**Figure 3:**
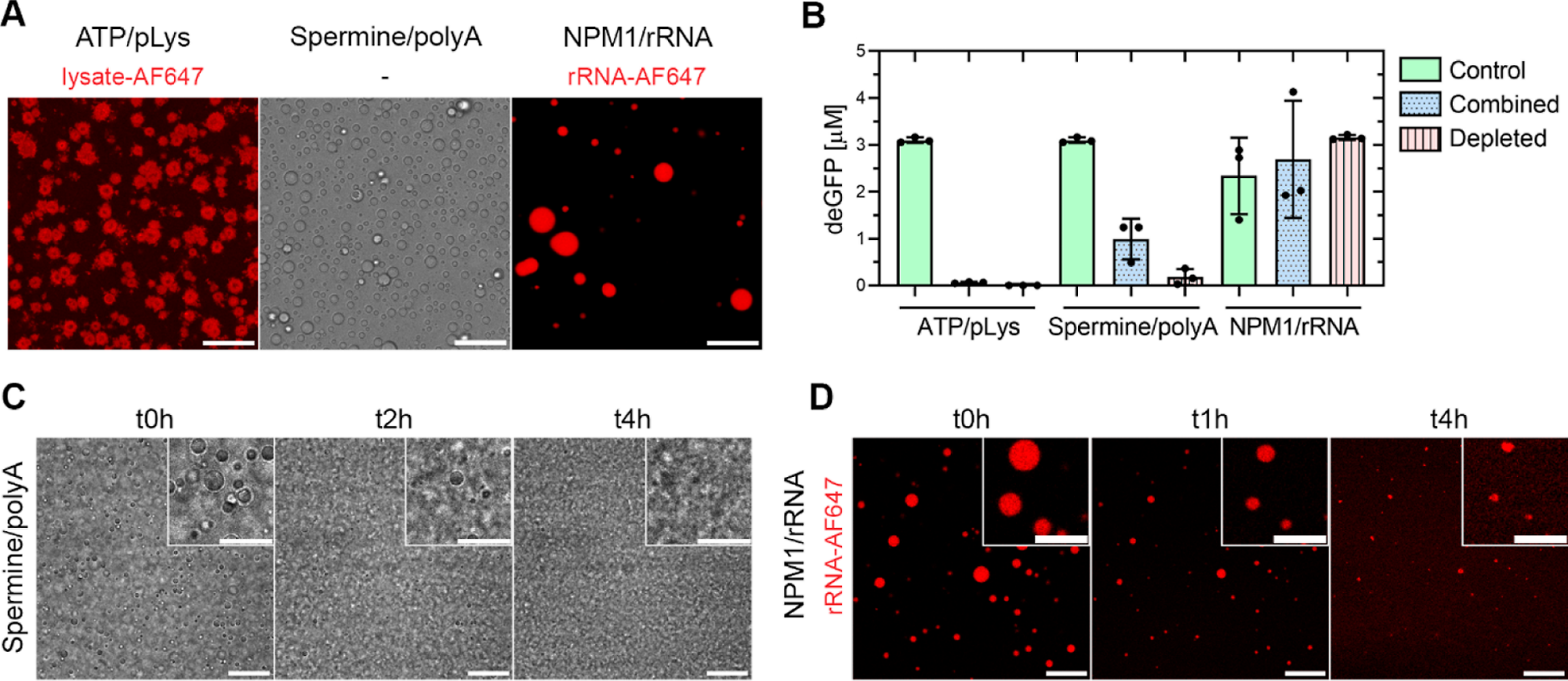
IVTT sequestration and stability of the three systems. (A) Confocal images of ATP/pLys, spermine/polyA, and NPM1/rRNA droplets in reduced ionic strength IVTT. ATP/pLys droplets were visualized by looking at AF647-labeled lysate. Scale bars 20 μm. (B) Endpoint deGFP concentration after >16 h expression inside 1.5 mL tubes incubated at 30 °C in the presence of ATP/pLys, spermine/polyA, or NPM1/rRNA droplets. Composition: ATP/pLys: 5 mM ATP, 5 mM pLys, 0.5 mM Tris-HCl pH 7.4. Spermine/polyA: 10 mM spermine, 1 mg/mL polyA, 0.5 mM Tris-HCl pH 7.4. NPM1/rRNA: 80 μM NPM1, 0.8 mg/mL rRNA, 0.5 mM Tris-HCl pH 7.4. For each condition, IVTT consisted of 10 mg/mL unlabeled cell lysate, 10 nM p70a-deGFP linear fragment, and reduced ionic strength feeding buffer as described in Table S5. Three different conditions: regular batch IVTT reaction (control), batch IVTT reaction in presence of droplets (combined), and batch IVTT reaction with droplets removed by centrifugation after an equilibration step to allow for sequestering of IVTT components into droplets. Error bars are standard deviations from N = 3 (C) transmission images of spermine/polyA coacervate droplets in the reduced ionic strength IVTT reaction mixture at 30 °C over time. Concentrations are the same as for spermine/polyA in 3A, with addition of 10 mg/mL unlabeled lysate and reduced ionic strength feeding buffer (see Table S5). Droplets dissolved and showed strong aggregation within 4 h. Scale bars 20 μm. Inset scale bars 10 μm. (C) Confocal fluorescence images of NPM1/rRNA droplets in the reduced ionic strength IVTT reaction mixture at 30 °C over time. Concentrations are the same as for NPM1/rRNA in 3A, with addition of 10 mg/mL unlabeled lysate and reduced ionic strength feeding buffer (see Table S5). Droplets dissolved almost completely within 4 h. Scale bars 20 μm. Inset scale bars 10 μm.

Based on these results, we hypothesized that some systems might be further stabilized by reducing the ionic strength of the IVTT reaction mixture. Particularly, the concentrations of amino acids, 3-PGA, K-glutamate, and Mg-glutamate were reduced, taking into account the effect on deGFP expression (Table S5, Figure S7). Using this reduced ionic strength IVTT mixture, we found that NPM1/rRNA and ATP/pLys droplets were indeed stabilized (Figure 3A). Thus, out of the eight initial systems, the four most promising remaining systems were GFP-K_72_/ssDNA, spermine/polyA, NPM1/rRNA, and ATP/pLys (Table S8).

### 2.3 Compatibility of Coacervates with Gene Expression

We next wanted to determine how the presence of ATP/pLys, spermine/polyA, NPM1/rRNA, or GFP-K_72_/ssDNA droplets influenced bulk deGFP expression. GFP-K_72_/ssDNA proved poorly compatible with fluorophore expression and will be discussed in the final section. Here, the p70a-deGFP linear DNA fragment concentration was standardized to 10 nM for all subsequent experiments. Direct comparison in terms of DNA concentration with the other expression systems described in Table S1 is difficult,^15−17^ as these systems employed different expression systems, different fluorescent proteins, and different DNA types (linear fragment versus plasmid), all of which influence protein expression.^5^ For our systems, we compared the endpoint deGFP concentration after 16 h of expression in a 20 μL reaction in a tube at 30 °C under three different conditions for each system (Figure 3B): (1) a control IVTT reaction without droplets present; (2) a combined system of IVTT and droplets; and (3) a depleted dilute phase. For the third condition, a combined sample of IVTT and droplets was prepared and incubated for 30–60 min at room temperature, after which droplets were removed by centrifugation, the dilute phase was isolated, and expression in the dilute phase was initiated by increasing the temperature to 30 °C. This condition served to test which systems depleted the IVTT reaction mixture of necessary components for expression. Complete droplet removal was confirmed via turbidity measurements (Figure S9). Importantly, we considered a system incompatible with in-droplet expression if there was no expression in the combined IVTT plus droplet condition.

As can be observed, ATP/pLys showed almost no expression in both the combined and depleted samples. This can be explained by the high charge density of the cationic polylysine, which binds small anionic species strongly, particularly the NTPs,^36^ leaving them unavailable for deGFP expression. For spermine/polyA, the combined samples showed a deGFP expression of 0.99 ± 0.35 μM, compared to nanomolar expression levels in the depleted sample. Finally, in the NPM1/rRNA system, the control, combined, and depleted samples all expressed deGFP in the range of 2–3 μM. The high expression in the depleted NPM1/rRNA sample is due to the highly specific interaction of RNA-binding domains with the negatively charged rRNA backbone.^25^ This means that, unlike ATP/pLys and spermine/polyA droplets, NPM1/rRNA droplets did not sequester IVTT components as strongly, leaving them available for protein expression after droplet removal.

### 2.4 Stability of Lysate-Containing Coacervate Droplets under Reaction Conditions

Initially, spermine/polyA and NPM1/rRNA could form droplets in the reduced ionic strength IVTT reaction mixture at room temperature (Figure 3A). As these systems proved to be compatible with bulk deGFP expression (Figure 3B), we next determined the stability of these droplets under reaction conditions at 30 °C using confocal microscopy. At this point, droplets were considered sufficiently stable if they did not dissolve within the first 6 h of expression, as a batch IVTT reaction usually reaches maximum product concentration within 6 h^5^ However, when spermine/polyA and NPM1/rRNA droplets were incubated in IVTT under reaction conditions, NPM1/rRNA droplets dissolved, while spermine/polyA droplets dissolved and showed aggregation within 4 h (Figure 3C,D). Here, spermine/polyA droplets were not labeled because the effect of a reduced ionic strength IVTT buffer on droplet morphology and stability was clear enough without additional labeling. The poor stability of NPM1/rRNA and spermine/polyA droplets can be explained by a combination of three factors. First, even under the minimal ionic strength IVTT conditions used in this study, the various systems are not far removed from their critical salt concentrations. Second, the charge composition and biomolecular composition of the IVTT reaction mixture changes due to enzymatic activity.^37−39^ Third, biomolecular components such as polyA, NPM1, and rRNA can be broken down by the various nucleases and proteases that are present in the *E. coli* lysate. This point is witnessed by the fact that NPM1/rRNA droplets can be stabilized to some extent by lowering the reaction temperature to 18 °C and adding a broad-spectrum RNAse inhibitor, Ribolock (Figure S10).

### 2.5 Protein Expression inside GFP-K_72_/ssDNA Droplets

**Figure 4:**
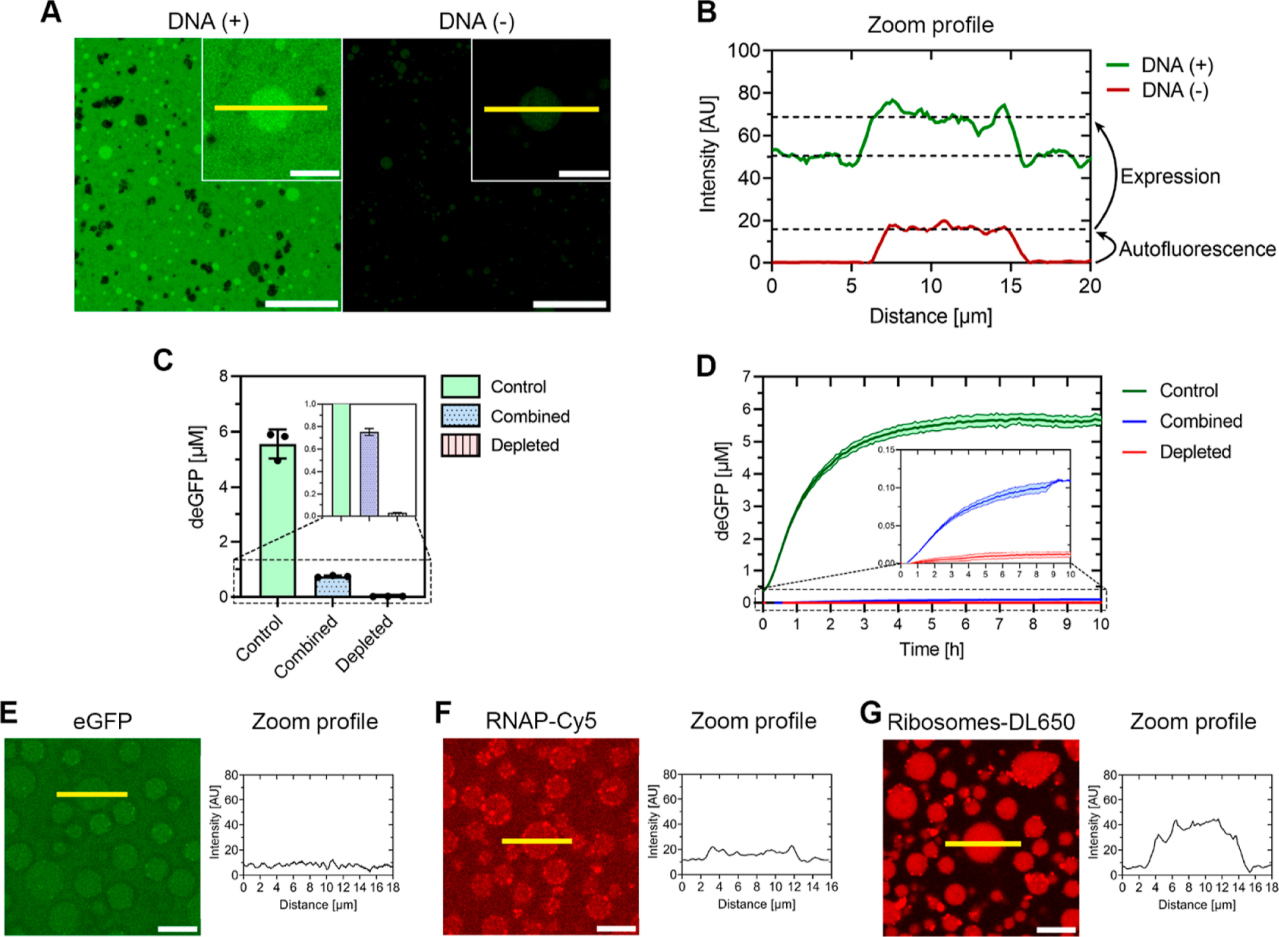
Expression of deGFP in GFP-K_72_-R97A/ssDNA droplets. (A) Expression of deGFP in GFP-K_72_-R97A/ssDNA droplets incubated for >16 h at 30 °C in a 1.5 mL tube, with or without the p70a-deGFP linear fragment. Inset contains zoom. Yellow lines indicate droplets in the intensity profile. Black spots are salt precipitates that form in the IVTT reaction over time. Scale bars 50 μm. Zoom scale bar: 20 μm. Final composition: 24 μM GFP-K_72_-R97A, 0.05 mg/mL ssDNA, 5 mM Tris-HCl pH 7.4, 1 mM per standard amino acid, 1.5 mM ATP, 1.5 mM GTP, 0.9 mM CTP, 0.9 mM UTP, 0.26 mM CoA, 0.33 mM NAD, 0.75 mM cAMP, 0.068 mM folic acid, 1 mM spermidine, 30 mM 3-PGA, 0.2 mg/mL *E. coli* tRNA, 40 mM K-glutamate, 6 mM Mg-glutamate, 2 wt % PEG8000, 10 nM p70a-deGFP linear DNA fragment, and 50 mM HEPES pH 8. The same composition was used in (B,C). For (E–G), the same GFP-K_72_-R97A/ssDNA concentrations were used, but without the added IVTT mixture. (B) Intensity profile of DNA (+) and DNA (−) zoomed droplets. (C) End point deGFP concentration after >16 h expression from 10 nM p70a-deGFP inside 1.5 mL tubes incubated at 30 °C under three conditions: regular batch IVTT reaction (control), batch IVTT reaction in presence of GFP-K_72_-R97A/ssDNA droplets (combined), and batch IVTT reaction with GFP-K_72_-R97A/ssDNA droplets removed by centrifugation after an equilibration step to allow for sequestering of IVTT components. *N* = 3. (D) Expression from the 10 nM p70a-deGFP linear fragment under three different conditions. Expression was followed using a plate reader at 30 °C in a 384-well microplate. *N* = 3. (E) Partitioning of 7.5 μM eGFP into GFP-K_72_-R97A/ssDNA droplets in the presence of IVTT after 30 min incubation at 30 °C. The yellow line indicates droplets in the intensity profile. Condensate composition same as in (A). Scale bar 10 μm. (F) Partitioning of 0.25 μM RNAP-Cy5 into GFP-K_72_-R97A/ssDNA droplets in the presence of IVTT after 30 min incubation at 30 °C. Yellow line indicates droplets in the intensity profile. Condensate composition same as in (A). Scale bar 10 μm. (G) Partitioning of 0.25 μM ribosomes-DL650 into GFP-K_72_-R97A/ssDNA droplets in the presence of IVTT after 30 min incubation at 30 °C. Yellow line indicates droplets in the intensity profile. Condensate composition same as in (A), but with added 5 mM Mg-glutamate for ribosome stability. Scale bar 10 μm.

Unlike spermine/polyA and NPM1/rRNA, GFP-K_72_/ssDNA remained stable under expression conditions for over 16 h (results not shown). However, this system proved incompatible with both deGFP and mmCherry expression. In both cases, the high concentration of GFP-K_72_, particularly inside the droplets, caused a high background signal and bleed-through even at low excitation levels (Figure S11). Thus, we constructed a non-fluorescent GFP-K_72_ mutant where the key arginine residue required for fluorophore maturation was mutated into an alanine residue (GFP-K_72_-R97A).^40,41^ This mutant behaved similarly to GFP-K_72_ and proved stable and compatible with deGFP expression (Figure S12). Moreover, GFP-K_72_-R97A/ssDNA droplets prepared in the presence of a full IVTT reaction mixture were more numerous and greater in size compared to pure GFP-K_72_-R97A/ssDNA droplets, again indicating robust IVTT uptake (Figure S12A,C). When GFP-K_72_-R97A/ssDNA/IVTT droplets were incubated with the deGFP gene added to the IVTT mixture, a clear fluorescence intensity difference with the negative control was observed (Figure 4A). This was also reflected in the intensity profiles of individual droplets (Figure 4B). Additionally, the same IVTT sequestering experiment as described above (Figure 3B) was performed with GFP-K_72_-R97A/ssDNA droplets (Figures 4C and S9D). The depleted condition showed no deGFP expression, while deGFP expression in the combined condition was retained at 0.75 ± 0.025 μM deGFP. Here, expression took place in a tube, which is an ideal condition for expression as there is a low total surface to volume ratio leading to less droplet loss due to adsorption to the walls, but a larger air-solution interface which generally results in higher expression levels. To follow the kinetics over time, we also expressed deGFP in a 10 μL reaction on a 384-well plate, which showed that the expression curve is similar to the control (Figure 4D).

To further support the notion that expression is taking place inside the droplets, we tested the capacity of GFP-K_72_-R97A/ssDNA droplets to partition purified eGFP, as well as two key IVTT components, namely, *E. coli* RNA polymerase labeled with Cyanine5 (RNAP-Cy5) and the fully assembled *E. coli* 70S ribosome labeled with DyLight650 (ribosomes-DL650). In each case, the relevant component was added to the cell lysate before the total system was assembled. After ∼30 min incubation, purified eGFP is only minimally enriched inside the GFP-K_72_-R97A/ssDNA droplets, with a partitioning coefficient (*K*_p_) of 1.15 ± 0.02 (Figure 4E). Crucially, >16 h incubation under reaction conditions does not increase the partitioning of eGFP into the droplets (*K*_p_ = 1.13 ± 0.02, Figure S13A). Moreover, if eGFP is added to GFP-K_72_-R97A/ssDNA droplets in the absence of an IVTT system, it is initially weakly excluded from the droplets (*K*_p_ = 0.66 ± 0.07, Figure S13B). Together, these observations also explain why an increase in fluorescence intensity was observed in both the dense and dilute phases, as deGFP was expelled into the dilute phase as expression progressed (Figure 4A). For RNAP-Cy5, we observed weak partitioning into the droplets after 30 min incubation under full reaction conditions (*K*_p_ = 1.55 ± 0.07, Figure 4F). Interestingly, incubation of RNAP-Cy5 in the absence of the IVTT reaction mixture showed a much higher partitioning of RNAP-Cy5 into the droplets (*K*_p_ = 12.03 ± 0.43, Figure S13C). Most likely, the interaction of RNAP with DNA and the numerous proteins present in the IVTT mixture prevents RNAP partitioning into the GFP-K_72_-R97A/ssDNA droplets as readily as when the IVTT mixture is absent.^42,43^ Another possibility is that other components in the IVTT mixture that partition into the condensate droplets compete with RNAP for client interaction-sites of the main condensate constituents. Finally, ribosomes-DL650 showed stronger partitioning into the droplets under full reaction conditions (*K*_p_ = 5.44 ± 0.18, Figure 4G). Thus, we have shown that the key enzymes involved in transcription and translation partition into GFP-K_72_-R97A/ssDNA droplets.

## 3. Conclusions

In summary, in our study we have systemically tested the compatibility of various phase-separating systems with in vitro transcription–translation. We have shown that deGFP can be expressed inside GFP-K_72_-R97A/ssDNA droplets. Overall, compatibility of this system with IVTT is not unsurprising, as GFP-ELP fusion proteins have previously been shown to be able to form intracellular compartments in live bacteria.^21^ Contrary to previous publications where related systems have been reported,^12,13^ we have achieved robust expressions levels, as is evidenced by micromolar level deGFP expression in our IVTT sequestering experiment (Figure 4). Moreover, we have shown that GFP-K_72_-R97A/ssDNA droplets can deplete an IVTT reaction mixture to the point where deGFP is no longer expressed in the dilute phase and that key components of transcription–translation partition into GFP-K_72_-R97A/ssDNA droplets (Figures 4 and S13). Additionally, we have provided rationales for the (lack of) compatibility of various systems with a lysate based IVTT system. The two key factors determining compatibility are the stability of the phase-separated droplets as a function of the ionic strength and charge composition of the IVTT reaction mixture, and the interaction strength between the droplet components and client molecules. For the latter, the interaction strength must not be too low to effectively partition all key IVTT components, as was the case for NPM1/rRNA, but also not too high, thereby interfering with transcription–translation, as was the case for ATP/pLys (Figures 3 and S4).

These two points are witnessed by the observation that deGFP expression in the combined GFP-K_72_-R97A/ssDNA/IVTT system is about eightfold lower than expression in a regular IVTT reaction (Figure 4C). There are several potential contributing factors that could explain this difference. First, while RNAP and ribosomes were shown to partition into the condensate droplets (Figures 4 and S13), critical small metabolites might not partition as strongly, reducing their in-droplet concentration. Second, condensate components could strongly sequester one of the many critical components of the IVTT mixture, such as RNAP, the ribosomes, or small charged metabolites such as the NTPs, thereby reducing their effective concentration and thus protein expression. This difference in sequestering strength also explains why spermine/polyA, which relies on a specific charge-based interaction for condensation, shows a strong reduction in expression between the combined and control samples, while NPM1/rRNA, which relies on domain-specific interaction for condensation, does not (Figure 3B). Third, another contributing factor could be the higher viscosity inside the GFP-K_72_-R97A/ssDNA droplets, which should primarily affect the diffusion of large biomolecules such as RNAP and the *E. coli* ribosomes. Fourth, alternatively, it could be the case that the linear DNA fragment is not taken up into the droplets, or that mRNA is degraded before protein expression can occur. However, several of systems explored in this work have a polynucleotide as the negatively charged component, which makes it unlikely that the linear DNA fragment is excluded from the condensate droplets. As for mRNA degradation, this is also unlikely as this is not a problem in standard IVTT batch reactions.^5,15,20,44^

Our results bear upon efforts to build coacervate-based proto- and synthetic cells. Much of the research on coacervation in synthetic cell research has focused on showing enhanced reactivity of simple reactions inside coacervate droplets.^26,45−47^ These chemically defined approaches are crucial for understanding the role coacervates might have played as protocells at the origins of life,^8−10,14^ and they are equally important in gaining understanding of how to functionalize such droplets as synthetic cell mimics.^13,14^ However, the extent to which condensation might have played a role in the origin of life and the extent to which biomolecular condensates might be functionalized is determined by the complexity of the reaction network that can in principle be incorporated. Our results provide a platform on which increasingly complex, biomolecular condensate-based synthetic cells can be built.

## 4. Materials and Methods

The materials and methods presented here are for quick reference. They are a heavily abridged version of the full materials and methods, which can be found in the Supporting Information.

### 4.1 Chemicals

All materials were purchased from Sigma-Aldrich unless otherwise specified. A detailed overview of the components used in this work can be found in the Supporting Information. Plasmids for the expression of p70a-deGFP and p70a-mmCherry were obtained from Daicel Arbor Biosciences and linearized using PCR (Table S15).

### 4.2 GFP-K_72_-R97A Plasmid Construction

A mutant version of GFP-K_72_ was constructed, with the key arginine amino acid residue at position 97 replaced by alanine.^40,41^ Site-directed mutagenesis was performed on the pET25-SfiI-GFP-ELP(K_72_) plasmid using a two-stage PCR reaction protocol (Table S15 for primers and constructs). Sanger sequencing was used to find the correct mutation among six colonies (Baseclear). The resulting plasmid was called pET25-SfiI-GFP-ELP(K_72_)-R97A.

### 4.3 GFP-K_72_ and GFP-K_72_-R97A Purification

GFP-K72 was purified as has been described previously.^22,48^ Overall, purification of GFP-K_72_ was similar to purification of the GFP-K_72_-R97A mutant. Briefly, *E. coli* BL21 (DE3) cells were transformed with pET25-SfiI-GFP-ELP(K_72_)-R97A. Large flasks of TB were inoculated and grown to *A*_600_ = 1.5, protein expression was induced with IPTG and carried out overnight at 20 °C. Cells were harvested, pelleted, and lyzed using a homogenizer. The lysate was clarified by centrifugation and the supernatant was loaded onto a 5 mL HisTrap FF (Cytiva). The eluted proteins were dialyzed overnight into size exclusion buffer, prior to loading onto a Superdex 200 16/600 size exclusion column (GE Healthcare) connected to an AKTA Basic FPLC (GE Healthcare). Fractions obtained were run on an SDS-PAGE gel to check for protein purity before pooling pure samples, and the protein concentration was determined using a NanoDrop One^C^. GFP-K_72_ and the mutant were flash-frozen and stored at −80 °C.

### 4.4 NPM1 Purification and Labeling

NPM1 was purified and labeled as described previously.^49^ Briefly, NPM1 was expressed in *E. coli* BL21 (DE3) cells. Cells were harvested and lyzed using a homogenizer, and debris was cleared using centrifugation. The supernatant was first purified using a His-Trap column (GE healthcare/Cytiva), concentrated, and purified further using size exclusion (Superdex 200, 16/600, GE healthcare). Protein samples were concentrated using Amicon-Ultra spin concentrators, and the concentration was determined using a NanoDrop One^C^. NPM1 was labeled using Alexa Fluor 488 C5 maleimide dye (ThermoFisher) according to the manufacturer’s protocol. Excess dye was removed through dialysis (Millipore, MWCO 3.5 kDa), and the concentration was determined using a NanoDrop One^C^. NPM1 was flash-frozen and stored at −80 °C.

### 4.5 *E. coli* Ribosomal RNA Purification and Labeling

rRNA was purified and labeled as described previously.^50^ Briefly, *E. coli* BL21 cells were harvested and homogenized, and debris was pelleted through centrifugation. The ribosome-containing supernatant was collected, and ribosomes were pelleted by ultracentrifugation. The ribosomes were resuspended and rRNA was isolated using standard phenol-chloroform extraction protocols. *E. coli* rRNA concentration was determined using a NanoDrop One^C^ and the 3′-end was labeled with Alexa Fluor 647-hydrazide (ThermoFisher) following Nelissen et al.^51^ After labeling, rRNA was purified using isopropanol purification and ethanol purification or using an Amicon spin filter (Millipore). An agarose gel was used to check dye removal and sample concentrations were determined using a NanoDrop.

### 4.6 eGFP Purification

*E. coli* BL21 (DE3) plus pET15b-His6-eGFP was grown overnight to a dense preculture. The full culture was grown at 30 °C, expression was induced with IPTG, and cultivation was continued overnight. Cells were harvested by centrifugation, dissolved in lysis buffer, and cells were lyzed using a probe sonicater (MSE Soniprep 150). Cell debris was removed by centrifugation and the supernatant was loaded onto a 5 mL His-Trap HP column. His-tagged eGFP was eluted, fractions were checked on SDS-PAGE, pooled, and dialyzed overnight. Any remaining precipitate after dialysis was removed by centrifugation. The concentration was determined spectroscopically. eGFP was flash-frozen and stored at −80 °C.

### 4.7 *E. coli* RNA Polymerase Purification and Labeling

*E. coli* RNA polymerase (RNAP) has been purified as has previously been described.^52^ Briefly, the RNAP holoenzyme (no σ factor) was overexpressed in *E. coli* BL21 (DE3), pelleted, and stored overnight at −80 °C. Pellets were redissolved in lysis buffer and cells were lyzed using a homogenizer. The supernatant was clarified twice using centrifugation and RNAP was isolated using a HisTrap HP column (Cytiva). Fractions were analyzed on SDS-PAGE and relevant fractions were pooled and purified using Heparin affinity chromatography on an AKTA Basic FPLC (GE Healthcare). Fractions were analyzed on SDS-PAGE and relevant fractions were combined. For labeling, RNAP was labeled using NHS-sulfoCy5 (Lumiprobe) following supplier instructions. The reaction took place for 4 h at room temperature and excess dye was removed through multiple rounds of dialysis. RNAP-Cy5 was stored in a 50 v/v % glycerol storage buffer at −20 °C. Before use, RNAP-Cy5 was dialyzed to a glycerol-free working buffer, and the concentration was determined using a Bradford assay.

### 4.8 *E. coli* Ribosome Purification and Labeling

*E. coli* BL21 (DE3) cells were grown to log phase and harvested using centrifugation. Cells were lyzed using a Mini-Beadbeater (BioSpec). Cellular debris was removed by centrifugation, and the supernatant was incubated at 37 °C for ribosome run-off. Clarification by centrifugation was repeated, and the supernatant was filtered (0.22 μm). Ribosomes in the supernatant were pelleted by ultracentrifugation. Ribosome pellets were dissolved, equilibrated to a gradient buffer, and separated using a 10–50% gradient of sucrose using ultracentrifugation. Gradients were harvested using a UV detector to isolate the 70S ribosomes.^53^ Ribosomes were subsequently pelleted by ultracentrifugation and dissolved in buffer, and the concentration was determined. Ribosomes were flash-frozen and stored at −80 °C.

*E. coli* 70S ribosomes were labeled using a protocol optimized from the literature.^54,55^ During the reaction, precipitate was removed by centrifugation. The supernatant containing ribosomes was concentrated in a centrifugal ultrafiltration device (Vivaspin 6, MWCO 30 kDa) and thoroughly washed to remove the excess of dye. The final concentration of labeled ribosomes was determined using a Nanodrop 1000 spectrophotometer (Isogen). The ribosomes were flash-frozen in liquid nitrogen and stored at −80 °C.

### 4.9 *E. coli* Lysate Labeling and IVTT Preparation

The in vitro transcription–translation system used in the work has been previously described by Sun et al.^56^ Some minor alterations were made. For lysate preparation, cell pellets were stored at −80 °C before lysis, cells were lyzed using a cell homogenizer, and S30B buffer contained 14 mM Mg-glutamate and 150 mM K-glutamate. Additionally, for the batch of lysate that was labeled with Alexa Fluor 647 NHS ester (ThermoFisher), a sodium phosphate buffer was used instead of Tris HCl. Feeding buffer was either prepared in total as described in Sun et al.,^56^ but with the added amino acid mixture as described in Caschera and Noireaux,^44^ or it was prepared in separate parts. For the feeding buffer components, the final concentrations can be found in Table S5. Lysate labeling was done following the instructions of the supplier, with an estimated dye concentration of <1:20 surface amines. Any remaining free dye was removed by dialysis (Slide-A-Lyzer, ThermoFisher). Lysate-AF647 was flash frozen and stored at −80 °C.

### 4.10 Confocal Microscopy

Microscopy was performed on a SP8x confocal microscope (Leica) or an SP8 liachroic confocal microscope (Leica). The SP8x uses a continuous white-light laser (WLL). Transmission light was collected using a PMT, while fluorescent light was collected using a Hybrid detector in counting mode. The SP8 liachroic has fixed laser lines at 488, 552, and 638 nm. Transmission light was collected using a PMT and fluorescent light was collected using a Hybrid detector in counting mode. Droplets were observed in a passivized chamber, either in an open configuration (18-well μ-Slide, Ibidi) or a closed configuration (two coverslips with a SecureSeal spacer sticker). All relevant surfaces were passivized with PLL-PEG. Image analysis was performed using ImageJ.

### 4.11 Lysate Mixing Order and Sequestration

Typically, coacervates (without lysate) were prepared by first mixing NaCl, Tris-HCl, MgCl_2_, MQ, and the desired type of negatively charged species in a microcentrifuge tube (0.5 mL, Eppendorf) at the required concentration, followed by the addition of positively charged species. The typical total volume was 20 μL. The final concentration of NaCl was 0 or 100 mM, and the final concentration of Tris-HCl and MgCl_2_ were 10–50 and 1–5 mM, respectively. For NPM1/rRNA coacervates, the concentrations of NaCl, Tris-HCl, and MgCl_2_ are 0 to 150, 10, and 0 to 5 mM, respectively. Mixing was done by gentle pipetting. To test the uptake a minimal amount of lysate-AF647 (0.25 mg/mL final), coacervates were prepared as described above, but the negatively charged component, positively charged component, and lysates were added in a different order to the relevant mixture of MQ, salts, and buffer. The mixing order taken as the starting point for subsequent experiments with increased lysate concentrations (Figures 2B and S4) can be found in Figure S3. Droplets were observed in a passivized glass chamber using confocal microscopy.

### 4.12 IVTT Sequestration

The effect of an increasing ionic strength of an IVTT solution was explored similarly to lysate sequestration. Coacervate systems components were mixed first and a dilution of both lysate and feeding buffer was added to the droplets. Here, it is important that the lysate and IVTT buffer are mixed in separately. Preparing a lysate/buffer mixture at increased concentrations (1.6×) leads to aggregation of the mixture (Figure S13). The effect of the IVTT mixture on droplet morphology and lysate uptake was observed using confocal microscopy in a passivized glass chamber. For the composition of the IVTT mixture, see Table S5.

### 4.12 Coacervate Compatibility with Expression

For the sequestering experiments, the various coacervate systems plus IVTT were prepared as described above. Each condition had a total reaction volume of 20 μL inside a 1.5 mL tube. Initially, GFP-K_72_/ssDNA, spermine/polyA, ATP/pLys, and NPM1/rRNA were explored in this way. For each system, three samples were taken. For the control sample, a regular IVTT positive control mixture was prepared. For the combined sample, the coacervate systems were mixed, and combined with lysate and feeding buffer as described above. After 30–60 min incubation at room temperature, 20 μL of droplets plus the IVTT mixture was taken from the tube. For the depleted sample, the remaining volume was centrifuged for 5 min at room temperature and 5000 RCF thereby pelleting the droplets. 20 μL of the supernatant (dilute phase) was transferred to a separate tube, taking care not to take along any pelleted material. For each sample, we tested the turbidity using absorbance measurements at 400 nm on a Tecan Spark plate reader (Figure S9). To determine the expression kinetics of each sample, conditions were repeated and 10 μL was loaded onto a clear bottom 384-well plate and deGFP expression was followed using a Tecan Spark plate reader set to 30 °C for 16 h. Each condition was tested in triplicate, using the minimal ionic strength feeding buffer composition (Table S5).

### 4.14 Stability in the IVTT Mixture under Reaction Conditions

The stability over time was determined by incubating the droplets with a full IVTT system at 30 °C. Here, coacervate droplets were formed first, after which the IVTT buffer was added, and lysate was added last. These were mixed by gentle pipetting. For the GFP-K_72_-R97A/ssDNA system, the lysate was added before the buffer (Figure S12). To determine droplet stability, droplets were loaded into a passivized glass chamber and followed using an SP8x confocal microscope with a temperature control box set to 30 °C over a period of 16 h. For the systems that were poorly or not stable in the original IVTT reaction mixture, a reduced ionic strength IVTT mixture was used.

### 4.15 Expression inside GFP-K_72_-R97A/ssDNA Droplets

Droplets were prepared from 24 μM GFP-K_72_-R97A, 0.05 mg/mL ssDNA, 5 mM Tris-HCl pH 7.5, 10 mg/mL lysate, and minimal ionic strength feeding buffer with the following changes: 1 mM amino acids, 30 mM 3-PGA, 6 mM magnesium glutamate, and no maltose. Feeding buffer either contained the 10 nM p70a-deGFP linear fragment or no DNA. The total reaction volume was 20 μL. Droplets were incubated in 1.5 mL tubes (Eppendorf) at 30 °C in a thermoshaker for ∼16 h. After incubation, the tubes were spun briefly to concentrate all material in the bottom of the tube. The droplets were harvested with a pipette and put in a closed, passivized glass chamber and observed using confocal microscopy.

### 4.16 RNAP, Ribosome, and eGFP Partitioning

Partitioning of *E. coli* RNAP-Cy5, *E. coli* ribosomes-DL650, and eGFP into GFP-K_72_-R97A/ssDNA droplets was determined using confocal microscopy. Coacervate droplets were prepared with RNAP-Cy5 (0.125 μM), ribosomes-DL650 (0.250 μM), or eGFP (7.5 μM) and incubated either in the tube or inside a closed chamber slide, at room temperature or 30 °C. After incubation, the droplets were imaged using either the SP8x or SP8 liachroic confocal microscope. The partitioning coefficient of each component into the droplets was determined by taking the average intensity of five droplets and dividing it by the average background intensity at five spots close to the droplets.

## Supporting Information Available

Detailed version of the methods, additional experimental data of the various compatibility experiments , and characterization of the GFP-K_72_-R97A mutant (PDF)

## Abbreviations

IVTT: in vitro transcription–translation
LLPS: liquid–liquid phase-separation
deGFP: deleted enhanced green fluorescent protein
ssDNA: single stranded deoxyribonucleic acid
tyRNA: torula yeast total ribonucleic acid
polyA: polyadenine
polyU: polyuracil
NPM1: nucleophosmin 1
rRNA: ribosomal RNA
ATP: adenosine triphosphate
pLys: polylysine
PDADMAC: poly(diallyldimethylammonium) chloride
PAA: polyacrylic acid
NTP: nucleoside triphosphate
3-PGA: 3-phosphoglyceric acid
RNAP-Cy5: RNA polymerase Cyanine5
DL650: DyLight650
AF488: Alexa Fluor 488
AF647: Alexa Fluor 647
SYBR Gold^57^: nucleic acid stain
