# Supplementary material for: In Vitro Transcription–Translation in an Artificial Biomolecular Condensate
Source: ACS Synth Biol. 2023 Jun 21;12(7):2004–14. doi: 10.1021/acssynbio.3c00069 (PMC10393115; doi:10.1021/acssynbio.3c00069)
Supplement: Supplementary file 1 — sb3c00069_si_001.pdf [file sb3c00069_si_001.pdf]

Supporting Information

In vitro transcription-translation in an artificial biomolecular condensate

Ludo L.J. Schoenmakers,[1] N. Amy Yewdall,[†],[1] Tiemei Lu,[†],[1] Alain A.M. André,[1] Frank. H.T. Nelissen,[1] Evan Spruijt,[1*] Wilhelm T.S. Huck[1*]

[1] Radboud University, Institute for Molecules and Materials, 6525 AJ Nijmegen, The Netherlands

Corresponding authors e-mail: w.huck@science.ru.nl; e.spruijt@science.ru.nl

Contents

1. Detailed methods S2
2. Supporting tables and figures S8
- S1: Overview of previous work on IVTT inside droplets S8
- S2: Overview of systems used in this study S8
- S3: Mixing order S9
- S4: Bacterial cell lysate uptake in seven systems S10
- S5: Final concentrations of charged species of various IVTT compositions S12
- S6: IVTT uptake in seven systems S13
- S7: deGFP expression as a function of IVTT composition S15
- S8: Compatibility overview S15
- S9: Turbidity measurements before and after droplet removal in four systems S16
- S10: NPM1/rRNA droplet stability under reaction conditions S17
- S11: Bleed-through from GFP-K_{72} S18
- S12: Phase-separation and fluorescence of GFP-K_{72}-R97A mutant S18
- S13: Partitioning into GFP-K_{72}-R97A/ssDNA droplets S19
- S14: Aggregation of lysate and feeding buffer at high concentrations S20
- S15: Primers and important sequence information S21
- S16: Spectrum of lysate-AF647 filter flow-through S22

1. Detailed methods

1.1 Chemicals. All materials were purchased from Sigma-Aldrich unless otherwise specified. For different types of coacervates, we used: spermine (TCI Europe N.V.), protamine sulfate salt from salmon (prot. sulf.), sodium citrate tribasic dihydrate (citrate), torula yeast ribonucleic acid (tyRNA), adenosine 5´-triphosphate disodium salt hydrate (ATP), polyadenylic acid potassium salt (polyA), polyuridylic acid potassium salt (polyU), poly-L-lysine hydrobromide (pLys, 15-30 kDa), poly(diallyl dimethylammonium chloride) (PDADMAC, 200-350 kDa, 20 wt% solution in H2O), and poly(acrylic acid) (PAA, 15 kDa, 35 wt% solution in H2O). Single-stranded DNA (ssDNA, $(ACTG)_{11}$, 44 bases) was purchased from Integrated DNA Technologies (IDT). Coacervates were prepared in solutions containing Tris(hydroxymethyl)-aminomethane hydrochloride (Tris HCl, pH 7.4), and sodium chloride (NaCl) and magnesium chloride hexahydrate ($MgCl_2 \cdot 6*H_2O$) as needed. For visualization purposes, SYBR Gold nucleic acid stain (10,000x concentration in DMSO) was purchased from ThermoFisher, and Cy3-labeled $polyT_{15}$ ($Cy3\text{-}T_{15}$) was purchased from IDT. Plasmids for the expression of p70a-deGFP and p70a-mmCherry were obtained from Daicel Arbor Biosciences (pTXTL series) and linearized using PCR (Table S15).

1.2 Stock solutions. The following chemicals were dissolved in MQ water (MQ, 18.2 MΩ cm) at the following typical stock concentrations: Tris-HCl (0.5 M, pH 7.4), $MgCl_2$ (50 mM), NaCl (3 M), ATP (50 mM), spermine (100 mM), prot. sulf (10 mg/ml), citrate (100 mM), pLys (50 mg/ml, 0.24 M in monomer unit), PAA (0.85 M in monomer unit), PDADMAC (50 mg/ml, 0.31 M in monomer unit) $GFP\text{-}K_{72}$ (140 µM), NPM1 (200 µM), and SYBR Gold. The following typical stock solutions were prepared in nuclease-free water: ssDNA $(ACTG)_{11}$ (0.46 mg/ml), polyA (6.4 mg/ml), polyU (10 mg/ml), torula yeast RNA (3.4 mg/ml), and the labeled DNA oligonucleotides $Cy3\text{-}T_{15}$ (100 nM). All the solutions were stored at −20 °C, except Tris-HCl, $MgCl_2$, and NaCl, which were stored at 4 °C. NPM1, rRNA, and their labeled variants, and eGFP, $GFP\text{-}K_{72}$, and $GFP\text{-}K_{72}$-R97A were stored at -80 °C.

1.3 $GFP\text{-}K_{72}$-R97A construction. A mutant version of $GFP\text{-}K_{72}$ was constructed, with the key arginine amino acid residue at position 97 replaced by alanine.[1,2] Site directed mutagenesis was performed on the pET25-SfiI-GFP-ELP(K_{72}) plasmid using a two-stage PCR reaction protocol (Table S15 for primers and constructs).[3] Briefly, 20 ng DNA plasmid was mixed with 10 µM of either primer, 2 mM dNTPs and 1X Pfu polymerase and the first PCR reaction was performed for 15 cycles, followed by mixing the two reactions together and then running the PCR for another 10 cycles. *E. coli* Top 10 cells were transformed with the Dpn1-treated plasmid reactions. Six colonies were selected for miniprep (QIAGEN) and sequenced to find the correct mutation using Sanger sequencing (Baseclear). The resulting plasmid was called pET25-SfiI-GFP-ELP(K_{72})-R97A.

1.4 $GFP\text{-}K_{72}$ and $GFP\text{-}K_{72}$-R97A purification. $GFP\text{-}K_{72}$ was purified as has been described previously.[4,5] Overall, purification of the $GFP\text{-}K_{72}$ was similar to purification of the $GFP\text{-}K_{72}$-R97A mutant. *E. coli* BL21 (DE3) cells were transformed with pET25-SfiI-GFP-ELP(K_{72})-R97A. Overnight cultures were used to inoculate large flasks of TB and cells were grown at 37 °C to A_{600} = 1.5, before protein expression was induced with 1 mM IPTG. Protein expression was carried out at 20 °C for 16 hours overnight, after which the cells were harvested by centrifugation. The pellet was resuspended in lysis buffer (10 mM Tris-HCl, pH 8, 300 mM NaCl, 25 mM imidazole) containing protease inhibitor tablets (Roche), 500 U Bezonase Nuclease and Bovine Pancreas RNAse A (VWR). The resuspended cells were lysed using a homogenizer at 1100 bar (Stansted 'Pressure Cell' Homogenizer SPCH-EP, Homogenising Systems LTD). The lysate was clarified at 35,000 RCF, 30 minutes, at 4 °C. The supernatant was loaded onto a 5 ml HisTrap FF (Cytiva). After loading, the column was washed with 50 ml of lysis buffer, and the His-tagged proteins were eluted using elution buffer (10 mM Tris-HCl, pH 8.0, 300 mM NaCl, 500 mM imidazole). The eluted proteins were dialyzed overnight into size exclusion buffer (20 mM Tris-HCl, pH 9, 300 mM NaCl), prior to loading onto a Superdex 200 16/600 size exclusion column (GE Healthcare) connected to an AKTA Basic FPLC (GE Healthcare). Fractionation of proteins was carried out at 1 ml/min and monitored at 280 nm and 260 nm. The fractions were analyzed on an SDS-PAGE gel to check for protein purity before pooling, and the protein concentration was determined using a

NanoDrop One^C using absorbance at 280 nm. The values were corrected using extinction coefficients based on protein sequence obtained from Protparam.

1.5 NPM1 purification and labeling. NPM1 was purified and labeled as described previously.[6] Recombinant poly-histidine tagged nucleophosmin-1 (NPM1) was expressed in *E. coli* BL21 (DE3) cells. Cells were harvested and lysed using a homogenizer (Stansted 'Pressure Cell' Homogenizer SPCH-EP, Homogenising Systems LTD) at 1100 bar in lysis buffer (10 mM Tris-HCl, pH 7.5, 300 mM NaCl, 20 mM imidazole, 1x protease inhibitor, 5 mM β-mercaptoethanol, and 0.1 mM PMSF). After pelleting at 20,000 RCF at 4 °C for 30 min, NPM1 was first purified using a His-Trap column (GE healthcare/Cytiva) using elution buffer (10 mM Tris-HCl, pH 7.5, 300 mM NaCl, 5 mM β-mercaptoethanol, and 500 mM imidazole). NPM1 was dialyzed overnight against SEC buffer (10 mM Tris-HCl, pH 7.5, 300 mM NaCl, 1 mM DTT) and concentrated to 5 ml using 10 kDa MWCO Amicon Ultra spin concentrators (Millipore). NPM1 was purified using a size exclusion column (Superdex 200, 16/600, GE healthcare) connected to an AKTA Basic FPLC pump (GE Healthcare) in SEC buffer. Protein samples were concentrated using Amicon-Ultra spin concentrators, and concentration was determined using a NanoDrop One^C and stored at -80 °C. NPM1 was labeled using AlexaFluor 488 C5 maleimide dye (ThermoFisher) according to the manufacturer's protocol. Excess dye was removed through dialysis (Millipore, MWCO 3.5 kDa) against SEC buffer, and the concentration was determined using the NanoDrop One^C.

1.6 *E. coli* ribosomal RNA purification and labeling. rRNA was purified and labeled as described previously.[7] *E. coli* BL21 (DE3) cells were harvested from 1 liter LB at A_{600} = 1.5 grown at 37 °C. Cells were pelleted and washed twice in lysis buffer (50 mM Tris-HCl pH 7.7, 60 mM potassium glutamate, 14 mM magnesium glutamate, 2 mM DTT). Cells were lysed at 1100 bar in a homogenizer (Stansted 'Pressure Cell' Homogenizer SPCH-EP, Homogenising Systems LTD) and cell debris was removed by centrifugation at 20,000 RCF for 25 minutes. Ribosomes were pelleted by ultracentrifugation for 3 hours at 50,000 RPM at 4 °C (Beckman Ti70.1 rotor). The ribosomes were resuspended in lysis buffer and rRNA was isolated using standard phenol-chloroform extraction protocols. *E. coli* rRNA concentration as determined using a NanoDrop One^C and the 3'-end was labeled with AlexaFluor647-hydrazide (ThermoFisher) following Nelissen *et al.*[8] After labeling, rRNA was purified using isopropanol purification and ethanol purification or using an Amicon spin filter (Millipore). An agarose gel was used to check dye removal and sample concentrations were determined using the NanoDrop One^C.

1.7 eGFP purification. *E. coli* BL21(DE3)/pLysS plus pET15b-His_6-eGFP was grown overnight at 37 °C in 25 ml of LB medium containing 100 µg/ml ampicillin and 34 µg/ml chloramphenicol while shaking. The dense-grown culture was used to inoculate 1 litre of the same medium and grown at 30 °C while shaking. At A_{600} = 1.5, eGFP expression was induced by adding IPTG to 1 mM and cultivation was continued overnight. Cells were harvested by centrifugation at 5000 RPM in a Beckman JA-10 rotor at 4 °C for 10 minutes and the pellet was stored overnight at -80 °C. Next day, the pellet was resuspended in ice-cold buffer A (25 mM sodium phosphate buffer, 300 mM NaCl, 5 mM β mercaptoethanol, 0.5 mM EDTA, 20 mM imidazole, pH 8.0) containing 0.5 mg/ml lysozyme and 0.1 mM PMSF and then sonicated on ice for 5 cycles of 1 minute at maximum amplitude (MSE Soniprep 150) with a 1 minute pause interval between each cycle. The lysate was centrifuged at 30,000 RCF in a Beckman JA-25.50 rotor at 4 °C for 45 minutes. The supernatant was loaded onto a 5 ml His-Trap HP column pre-equilibrated with buffer A in a cold room. The column was successively washed with 25 ml of buffer A and 25 ml of buffer A plus 50 mM imidazole. His-tagged eGFP was eluted with buffer A plus 300 mM imidazole and collected in fractions of 1 ml. Fractions were checked on SDS-PAGE and those containing pure eGFP were pooled, dialyzed overnight at 4 °C against 300 volumes of dialysis buffer (25 mM sodium phosphate buffer, 0.5 mM DTT, 0.1 mM EDTA, pH 8.0). Next day, the dialysis buffer was refreshed and dialysis continued for another 8 hours. The purified eGFP isolate was then centrifuged at 15,000 RCF at 4 °C for 1 minute to remove any formed precipitate, concentrated to 100 µM (fluorophore-based) in a 10 kDa MWCO Vivaspin 6 ml ultrafiltration device (Sartorius), aliquoted, flash frozen and stored at -80 °C.

1.8 *E. coli* RNA polymerase purification and labelling. *E. coli* RNA polymerase (RNAP) has been purified as has described previously.[9] A preculture of *E. coli* BL21 (DE3) with plasmid PI9000 (RNAP holoenzyme) in LB plus Ampicillin was grown overnight. The preculture was used to inocculate 2x 600 ml LB plus Amp, which was grown to A_{600} = 0.75 at 37 °C, 220 RPM. Cells were harvested by centrifugation at 6000 RCF, 10 min, 4 °C. Supernatant was discarded and pellets were stored at -80 °C. The next day, pellets were redissolved in lysis buffer (50 mM sodium phosphate pH 7, 500 mM NaCl, 5 v/v% glycerol, 0.1 mM PMSF). Cells were lysed at 1100 bar using a homogenizer (Stansted 'Pressure Cell' Homogenizer SPCH-EP, Homogenising Systems LTD) and supernatant was clarified twice using centrifugation at 29,000 RCF for 30 minutes at 4 °C. RNAP was isolated using a HisTrap HP column (Cytiva) with elution buffer (50 mM sodium phosphate pH 7, 500 mM NaCl, 5 v/v% glycerol, 0.1 mM PMSF, 250 mM imidazole). Fractions were analyzed on SDS-PAGE and relevant fractions were pooled and dialyzed in 3.5 kDa MWCO dialysis tubing against 100 volumes of buffer AB5 (50 mM sodium phosphate pH 7, 75 mM NaCl, 5 v/v% glycerol, 0.1 mM PMSF, 0.5 mM EDTA pH 8, 1 mM DTT) at 4 °C overnight. Next day, RNAP was purified on a 5 ml Heparin column using an AKTA Basic FPLC (GE Healthcare) running a 0-100% gradient of buffer A (50 mM sodium phosphate pH 7, 5 v/v% glycerol, 0.1 mM PMSF, 0.5 mM EDTA pH 8, 1 mM DTT) to buffer B (50 mM sodium phosphate pH 7, 1.5 M NaCl, 5 v/v% glycerol, 0.1 mM PMSF, 0.5 mM EDTA pH 8, 1 mM DTT). RNAP was eluted between 30-45 mS/cm. Fractions were analyzed on SDS-PAGE and relevant fractions were combined.

For labeling, relevant RNAP fractions were dialyzed against 100 volumes of labeling reaction buffer (100 mM NaCl, 10 mM sodium phosphate buffer pH 8, 0.1 mM EDTA, 0.1 mM DTT, 5 v/v% glycerol) in 3.5 kDa MWCO dialysis tubing overnight at 4 °C. Next morning, RNAP was dialyzed against fresh reaction buffer for 4 more hours and added directly to 1:5 RNAP:NHS-sulfoCy5 (Lumiprobe) molar ratio of dry NHS-sulofCy5 powder and a magnetic stirring bar. Conjugation took place at room temperature, for 4 hours, under continuous movement, and keeping the pH at 8 using sodium phosphate buffer. After 4 hours, 5 mM Tris-HCl pH 8 was added to react with remaining free NHS-sulfoCy5 for 1 hour at room temperature. The reaction solution was dialyzed in 3.5 kDa MWCO dialysis tubing against 1000 volumes of RNAP storage buffer (100 mM NaCl, 10 mM sodium phosphate buffer pH 7.5, 0.1 mM EDTA, 0.1 mM DTT, 50 v/v% glycerol) at 4 °C. Next day, the RNAP storage buffer was replenished and dialyzed for 4 more hours. RNAP-Cy5 was aliquoted and stored at -20 °C. Before use, RNAP-Cy5 was dialyzed for 5-6 hours in a 6-8 kDa MWCO D-Tube Dialyzer (Millipore) against 1000 volumes of RNAP working buffer (20 mM NaCl, 5 mM sodium phosphate buffer pH 7) at 4 °C and the RNAP-Cy5 concentration was determined using a Bradford assay.

1.9 *E. coli* ribosome purification and labeling. *E. coli* BL21 (DE3) cells at A_{600} = 1.5 were harvested by centrifugation at 10,000 RCF for 10 minutes at 4 °C. Cells were lysed in monosome gradient buffer (20 mM Tris-HCl pH 8.0, 10 mM $MgCl_2$, 140 mM KCl, 1 mM DTT, 0.1 mM EDTA, 20 U/ml Superase-In) using a Mini-Beadbeater (BioSpec). Cellular debris was removed by centrifugation at 33,000 RCF for 30 minutes at 4 °C and the supernatant was incubated at 37 °C for 80 minutes (ribosome run-off). Supernatant centrifugation was repeated and filtered using a 0.22 µm pore-size filter. Ribosomes in the supernatant were pelleted by ultracentrifugation for 3 hours at 211,000 RCF at 4 °C in a Beckman Ti70.1 rotor. Ribosome pellets were dissolved overnight at 4 °C in a small volume of monosome gradient buffer using a 10 RPM and 30° angle shaking program on a rotating mixer (no full rotations). Approximately 200 A_{260} units of *E.coli* BL21 ribosomes were adjusted to 1 ml with monosome gradient buffer and loaded onto a 10-50% gradient of sucrose in monosome buffer in SW-28 tubes (Beckman) and centrifuged in a Beckman SW28-rotor for 20 hours at 58,000 RCF, at 4 °C. Gradients were harvested at 4 °C using a glass capillary connected to a peristaltic pump and a UV detector and the peak containing the 70S ribosomes[10] was fractionated in portions of ~1 ml. Ribosomes were subsequently pelleted by ultracentrifugation in a Ti70.1 rotor for 3 hours at 211,000 RCF, at 4°C. The pellet was dissolved in 500 µl of monosome buffer, the concentration determined, and flash frozen in liquid nitrogen and stored at -80 °C.

E. coli 70S ribosomes were labeled using a protocol optimized from the literature.[11,12] The labeling reaction was conducted in a buffer with the following composition: 50 mM Tris-HCl, pH 7.6, 15 mM $MgCl_2$, 100 mM NH_4Cl and 6 mM β-mercaptoethanol, containing 4.8 µM of ribosomes and 250 µM of DyLight650 NHS-ester (ThermoFisher). The reaction mixture was incubated at 37 °C for 30 minutes and precipitate was

removed by centrifugation for 1 minute at 10,000 RPM in a tabletop centrifuge. The supernatant containing ribosomes was concentrated in a centrifugal ultrafiltration device (Vivaspin 6, MWCO 30 kDa) and thoroughly washed with the labeling buffer to remove the excess of dye. The final concentration of labeled ribosomes was 6.3 µM and the DyLight650 concentration was 23.3 µM as determined using a Nanodrop 1000 spectrophotometer (Isogen). This corresponds to ~4 labels per ribosome. The ribosomes were flash frozen in liquid nitrogen and stored at -80 °C until further use.

1.10 *E. coli* lysate labeling and IVTT preparation. The *in vitro* transcription-translation system used in the work has been previously described by Sun *et al*.[13] Some minor alterations were made. For lysate preparation, cell pellets were stored at -80 °C before lysis, cells were lysed using a cell homogenizer (Stansted 'Pressure Cell' Homogenizer SPCH-EP, Homogenising Systems LTD) at 1100 bar, and S30B buffer contained 14 mM magnesium glutamate and 150 mM potassium glutamate. Additionally, for the batch of lysate that was labeled with Alexa Fluor 647 NHS ester (ThermoFisher), a sodium phosphate buffer at pH 7.5 was used instead of Tris-HCl. Labeling was done following the instructions of the supplier, with an estimated molar dye concentration of <1:20 surface amines. Any remaining free dye was reacted with Tris-HCl and removed through overnight dialysis at 4 °C against 500 volumes of S30B buffer in 10 kDa MWCO dialysis cassette (Slide-A-Lyzer, ThermoFisher), followed by another 8 hours dialysis against 500 volumes of fresh S30B buffer. A small volume of labeled lysate was diluted 1:1 in S30B buffer and passed over a 10 kDa MWCO spin filter (Merck Millipore). A spectrum of the flow-through was taken (NanoDrop OneC) to check for remaining free dye (Fig. S16). Lysate-AF647 was aliquoted, flash frozen in liquid nitrogen, and stored at -80 °C.

Feeding buffer was either prepared in total as described in Sun *et al.*,[13] but with the added amino acid mixture as described in Caschera and Noireaux,[14] or it was prepared in separate parts. For the partial preparation, the amino acids solution (AA), energy solution (ES) without 3-phosphoglyceric acid (3-PGA), and with 3-PGA were added separately to the IVTT reaction mixture. The typical lysate protein final concentration for an IVTT reaction was 10 mg/ml. For the feeding buffer components, the final concentrations can be found in Table S5. DNA was added as a linear fragment of p70a-deGPF or p70a-mmCherry.

1.11 Confocal microscopy. Microscopy was performed on a SP8x confocal microscope (Leica) or an SP8 liachroic confocal microscope (Leica). The SP8x uses a continuous white-light laser (WLL), with typical excitation wavelengths of 488 nm (GFP-K_{72}, eGFP, NPM1, SYBR Gold), 552 nm (Cy3-T_{15}), and 647 nm (labeled lysate, mmCherry, rRNA-AF647). Transmission light was collected using a PMT, while fluorescent light was collected using a Hybrid detector in counting mode. Typical emission collection ranges were 496-550 nm (GFP-K_{72}, eGFP, NPM1, SYBR Gold), 565-580 nm (Cy3-T_{15}), 650-800nm (labeled lysate, IVTT, mmCherry, rRNA). The SP8 liachroic has fixed laser lines at 488 nm, with the same collection ranges as the SP8x. Transmission light was collected using a PMT and fluorescent light was collected using a Hybrid detector in counting mode. For visualization, labeled coacervate components have been described above. Non-labeled components were visualized by adding either SYBR Gold (PDADMAC/PAA, APT/pLys, prot. sulf./citrate) or Cy3-T_{15} (spermine/polyA, spermine/polyU). Droplets were observed in a passivized chamber. For an open chamber, a plasma-cleaned 18-well µ-Slide (Ibidi) was incubated with 0.1 mg/ml PLL-PEG for 3 hours minimum, washed with MQ, and dried with nitrogen gas. For a closed chamber, a two-sided spacer sticker (SecureSeal 25x25 mm, 0.12 mm depth) was mounted onto a coverslip (26x76 mm, #1.5 Epredia), plasma-cleaned, and incubated with 0.1 mg/ml PLL-PEG for 3 hours minimum, cleaned with MQ, and dried with nitrogen gas. After loading the sample, the chamber was closed with a round glass coverslip (Ø 26 mm, VWR). Image analysis was performed using ImageJ.

1.12 Lysate mixing order and sequestration. Typically, coacervates (without lysate) were prepared by first mixing NaCl, Tris-HCl, $MgCl_2$, MQ and the desired type of negatively charged species such as citrate, polyU, polyA, RNA, ssDNA, ATP or PAA in a microcentrifuge tube (0.5 ml, Eppendorf) at the required concentration, followed by the addition of positively charged (i.e. the relevant part) prot. sulf., spermine, GFP-K_{72}, NPM1, pLys and PDADMAC from their respective stock solutions. The total volume was 20 µL. The

final concentration of NaCl is 0 or 100 mM and the final concentration of Tris-HCl and $MgCl_2$ are 50 mM and 5 mM, respectively. For NPM1/rRNA coacervates, the concentration of NaCl, Tris-HCl, and $MgCl_2$ are 0 to 150 mM, 10 mM, and 0 to 5 mM, respectively. Mixing was done by gentle pipetting. To test the uptake a minimal amount of lysate-AF647 (0.25 mg/ml final), coacervates were prepared as described above, but the negatively charged component, positively charged component, and lysate were added in a different order to the relevant mixture of MQ, salts, and buffer. The mixing order taken as the starting point for subsequent experiments can be found in Fig. S3. Droplets were observed in a passivized glass chamber using confocal microscopy.

1.13 IVTT sequestration. The effect of an increasing ionic strength of an IVTT solution was explored similarly to lysate sequestration. Coacervate systems components were mixed first and a dilution of the full IVTT mixture (lysate plus feeding buffer) was added to the droplets. For the composition of the IVTT mixture, see Table S5. Here, it is important that the lysate and IVTT buffer are mixed in separately. Preparing a lysate/buffer mixture at increased concentrations (1.6x) leads to aggregation of the mixture (Fig. S13). The effect of the IVTT mixture on droplet morphology and lysate uptake was observed using confocal microscopy in a passivized glass chamber.

1.14 Coacervate compatibility with expression. For the sequestering experiments, the various coacervate systems plus IVTT were prepared as described above. For each system, three samples were taken. For the control sample, a regular IVTT positive control mixture was prepared using the same batch of components used for the other samples. For the combined sample, the coacervate systems were mixed and combined with lysate and feeding buffer as described above. After 50 minutes incubation at room temperature, a small volume of droplets plus IVTT mixture was taken from the tube. For the depleted sample, the remaining volume was centrifuged for 5 minutes at room temperature and 5000 RCF in a tabletop centrifuge thereby pelleting the droplets (Fig. S9). A small volume of supernatant (dilute phase) was taken from the tube. For each sample, we tested the turbidity using absorbance measurements at 400 nm on a Tecan Spark plate reader. To determine the expression of each sample, 10 µl volumes of each sample was loaded onto a clear bottom 384-well plate and deGFP expression was followed using a Tecan Spark plate reader set to 30 °C for 16 hours. Each condition was tested in triplicate.

1.15 Stability in IVTT mixture under reaction conditions. The stability over time was determined by incubating the droplets with a full IVTT system at 30 °C. Initially, GFP-K_{72}/ssDNA, spermine/polyA, ATP/pLys, and NPM1/rRNA were explored in this way. Here, coacervate droplets were formed first, after which the IVTT buffer was added, and lysate was added last. These were mixed by gentle pipetting. For the GFP-K_{72}-R97A/ssDNA system, the lysate was added before the buffer (Fig. S12). To determine droplet stability, droplets were loaded into a passivized glass chamber and followed using an SP8x confocal microscope with a temperature control box set to 30 °C over a period of 16 hours. For the systems that did not prove stable in an IVTT reaction mixture, a reduced ionic strength IVTT mixture was used. For several systems, the effect of adding 1 U inorganic pyrophosphatase (IPP), 0.1 mM PMSF protease inhibitor, and/or 1 U Ribolock RNAse inhibitor (ThermoFisher) on stability was also explored.

1.16 Expression inside GFP-K_{72}-R97A/ssDNA droplets. Droplets were prepared from 24 µM GFP-K_{72}-R97A, 0.05 mg/ml ssDNA, 5 mM Tris-HCl pH 7.5, 10 mg/ml lysate, and minimal ionic strength feeding buffer with the following changes: 1 mM amino acids, 30 mM 3-PGA, 6 mM magnesium glutamate, and no maltose. Feeding buffer either contained 10 nM p70a-deGFP linear fragment (DNA(+)) or no DNA ((DNA(-)). The total reaction volume was 20 µl. Droplets were incubated in 1.5 ml tubes (Eppendorf) at 30 °C in a thermoshaker for ~16 hours. After incubation, the tubes were spun briefly to concentrate all material in the bottom of the tube. The droplets were harvested with a pipette and put in a closed, passivized glass chamber and observed using confocal microscopy.

1.17 RNAP, ribosome, and eGFP partitioning. Partitioning of *E. coli* RNAP-Cy5, *E. coli* ribosomes-DL650, and eGFP into GFP-K_{72}-R97A/ssDNA droplets was determined using confocal microscopy. Coacervate droplets

were prepared in 1.5 ml tubes, RNAP-Cy5 (0.125 µM), ribosomes-DL650 (0.250 µM), and eGFP (7.5 µM) were added and incubated either in the tube or inside a closed chamber slide under various conditions. After incubation, the droplets were imaged using either the SP8x or SP8 liachroic confocal microscopes. The partitioning coefficient of each component into the droplets was determined by taking the average intensity of five droplets and dividing it by the average background intensity at five spots close to the droplets. All images were analyzed using ImageJ, including profile intensity measurements.

References

1. Wood, T. I. et al. Defining the role of arginine 96 in green fluorescent protein fluorophore biosynthesis. Biochemistry 44, 16211–16220 (2005).
2. Barondeau, D. P., Putnam, C. D., Kassmann, C. J., Tainer, J. A. & Getzoff, E. D. Mechanism and energetics of green fluorescent protein chromophore synthesis revealed by trapped intermediate structures. Proc. Natl. Acad. Sci. U. S. A. 100, 12111–12116 (2003).
3. Wang, W. & Malcolm, B. A. Two-Stage PCR Protocol Allowing Introduction of Multiple Mutations, Deletions and Insertions Using QuikChangeTM Site-Directed Mutagenesis. BioTechniques 26, 680–682 (1999).
4. Pesce, D., Wu, Y., Kolbe, A., Weil, T. & Herrmann, A. Enhancing cellular uptake of GFP via unfolded supercharged protein tags. Biomaterials 34, 4360–4367 (2013).
5. Brinke, E. te et al. Dissipative adaptation in driven self-assembly leading to self-dividing fibrils. Nat. Nanotechnol. 13, 849 (2018).
6. André, A. A. M., Yewdall, N. A. & Spruijt, E. Crowding-induced phase separation and gelling by co-condensation of PEG in NPM1-rRNA condensates. Biophys. J. 122, 397–407 (2023).
7. Yewdall, N. A. et al. ATP:Mg2+ shapes material properties of protein-RNA condensates and their partitioning of clients. Biophys. J. 121, 3962–3974 (2022).
8. Nelissen, F. H. T. et al. Improving Breast Cancer Treatment Specificity Using Aptamers Obtained by 3D Cell-SELEX. Pharmaceuticals 14, 349 (2021).
9. Svetlov, V. & Artsimovitch, I. Purification of Bacterial RNA Polymerase: Tools and Protocols. in Bacterial Transcriptional Control: Methods and Protocols (eds. Artsimovitch, I. & Santangelo, T. J.) 13–29 (Springer, 2015). doi:10.1007/978-1-4939-2392-2_2.
10. Christodoulou, J. et al. Heteronuclear NMR investigations of dynamic regions of intact Escherichia coli ribosomes. Proc. Natl. Acad. Sci. 101, 10949–10954 (2004).
11. Blanchard, S. C., Kim, H. D., Gonzalez, R. L., Puglisi, J. D. & Chu, S. tRNA dynamics on the ribosome during translation. Proc. Natl. Acad. Sci. 101, 12893–12898 (2004).
12. Thiele, J. et al. DNA-functionalized hydrogels for confined membrane-free in vitro transcription/ translation. Lab. Chip 14, 2651–2656 (2014).
13. Sun, Z. Z. et al. Protocols for implementing an Escherichia coli based TX-TL cell-free expression system for synthetic biology. J. Vis. Exp. JoVE e50762 (2013) doi:10.3791/50762.
14. Caschera, F. & Noireaux, V. Synthesis of 2.3 mg/ml of protein with an all Escherichia coli cell-free transcription-translation system. Biochimie 99, 162–168 (2014).

2. Supporting tables and figures

Reference	Droplet system	Expression system	Expression source	Protein	Total expression (up to)	Droplet phase expression (up to)	In-droplet expression (up to)
Sokolova *et al*. 2013[14]	Phase-separated PEG8000	Homemade *E. coli*-based IVTT[34,35]	Plasmid: pRSET5d-UTR1-eGFP-Del6-229[34]	eGFP	Not applicable	Not determined	350 nM
Tang *et al*. 2015[15]	Carboxymethyl-dextran/ polylysine (CM-dextran/pLys)	Commercial Expressway Cell-Free *E. coli* Expression System	Plasmid: pEXP5-NT/mCherry[15]	mCherry	10 nM	Not applicable	Not determined
Xu *et al*. 2022[16]	poly(diallyldimethylammonium chloride)/adenosine triphosphate (PDADMAC/ATP)	Lysate: in-droplet lysed *E. coli* cells. Buffer: commercial Expressway Mini Cell-Free Expression System/	Plasmid: pEXP5-NT/ deGFP[16]	deGFP	10 nM	10 nM	Not determined
This study	GFP-K_{72}-R97A/ssDNA	Homemade *E. coli*-based IVTT[34,35]	Plasmid: pTxTl-p70a-deGFP (Arbor Bioscience)	deGFP	0.75 µM	Not applicable	0.75 µM

Table S1. Overview of previous work on IVTT inside droplets. Also including the results from this work. Total expression = full systems of droplets and supernatant. Droplet phase expression = expression in droplet phase separated from dilute phase by centrifugation. In-droplet expression = expression levels of fluorescent protein inside actual droplets.

#	Component A	Component B	Pairing
1	GFP-K_{72}	Single-stranded DNA (ssDNA)	Cationic polymer/ single-stranded polynucleotide
2	GFP-K_{72}	Torula yeast RNA (tyRNA)	Cationic polymer/single-stranded polynucleotide
3	Spermine	Polyadenine (polyA)	Polyamine/single-stranded polynucleotide
4	Spermine	Polyuracil (polyU)	Polyamine/single-stranded polynucleotide
5	Nucleophosmin 1 (NPM1)	Ribosomal RNA (rRNA)	Protein/single-stranded polynucleotide
6	Polylysine (pLys)	Adenosine triphosphate (ATP)	Cationic polymer/nucleoside triphosphate
7	Protamine sulfate (Prot. sulf.)	Citrate	Protein/multivalent anion
8	Poly(diallyldimethylammonium) (PDADMAC)	Polyacrylic acid (PAA)	Anionic synthetic polymer/cationic synthetic polymer

Table S2. Overview of systems used in this work. References to various systems can be found in the main text.

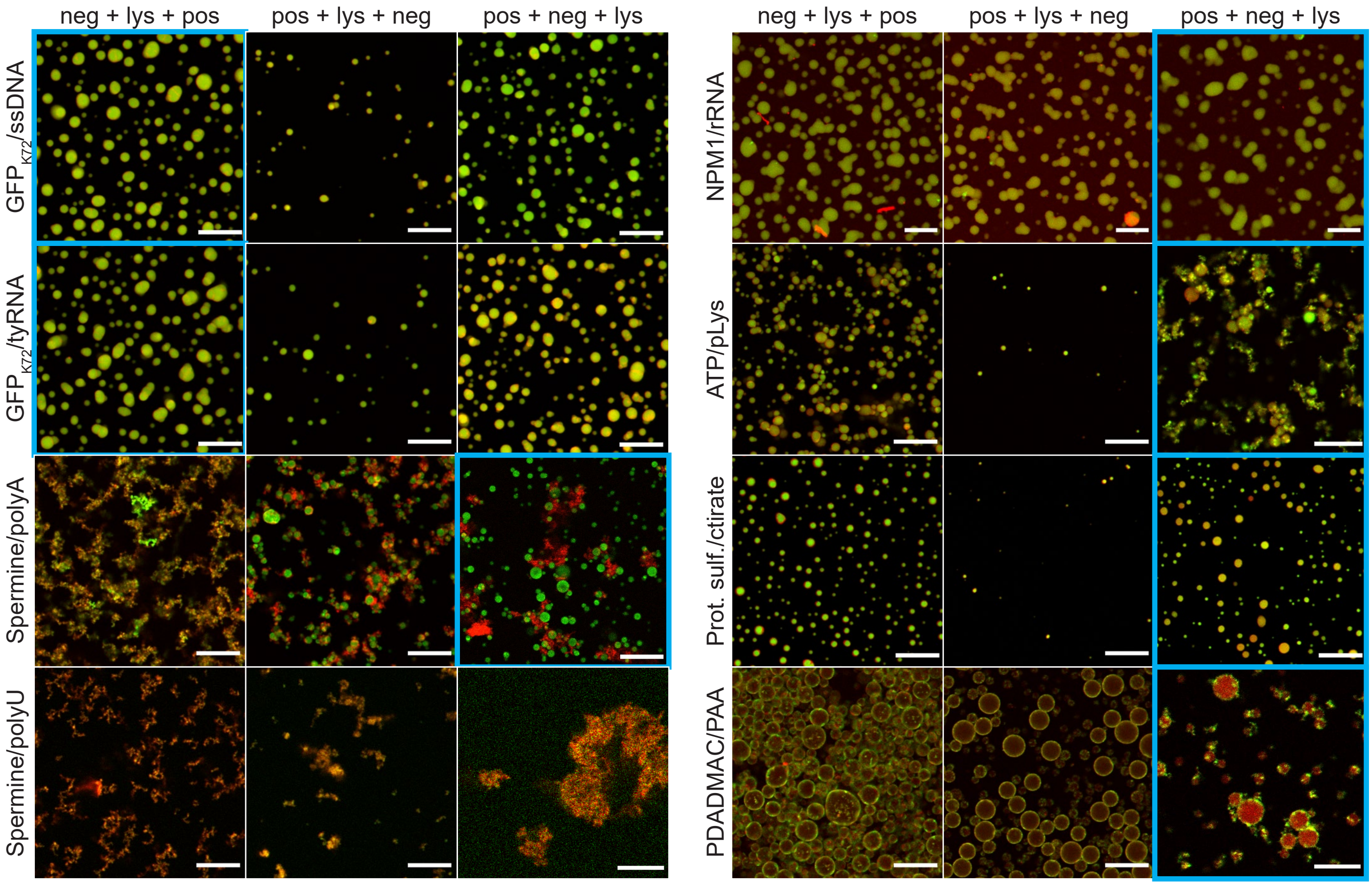

Figure S3. Mixing order. Mixing order-dependent effect of bacterial cell lysate on phase separation. Mixing order used as the basis for the lysate and IVTT experiments in Fig. 2, Fig. S4, and Fig. S6 have a blue border. All images are green/red channel overlays. Green channel = coacervate component/coacervate dye. Red channel = lysate-AF647. Labels used: GFP-K_{72}/ssDNA and GFP-K_{72}/tyRNA, GFP-K_{72}; spermine/polyA and spermine/polyU, Cy3-T_{15}; NPM1/rRNA, NPM1-AF488; ATP/pLys, PDADMAC/PAA, and prot. sulf./citrate, SYBR Gold. Composition of each system: 12 µM GFP-K_{72}, 0.025 mg/ml ssDNA, 5 mM $MgCl_2$, 50 mM Tris-HCl pH 7.4; 12 µM GFP-K_{72}, 0.3 mg/ml tyRNA, 5 mM $MgCl_2$, 50 mM Tris-HCl pH 7.4; 10 mM spermine, 1 mg/ml polyA, 1 mM $MgCl_2$, 10 mM Tris-HCl pH 7.4; 10 mM spermine, 1 mg/ml polyU, 1 mM $MgCl_2$, 10 mM Tris-HCl pH pH 7.4; 20 µM NPM1, 0.2 mg/ml rRNA, 150 mM NaCl, 10 mM Tris-HCl pH pH 7.4; 5 mM ATP, 5 mM pLys, 5 mM $MgCl_2$, 100 mM NaCl, 10 mM Tris-HCl pH pH 7.4; 3 mg/ml prot. sulf., 5 mM citrate, 5 mM $MgCl_2$; 20 mM PDADMAC, 20 mM PAA, 100 mM NaCl, 10 mM Tris-HCl pH pH 7.4; .Scale bars 20 µm.

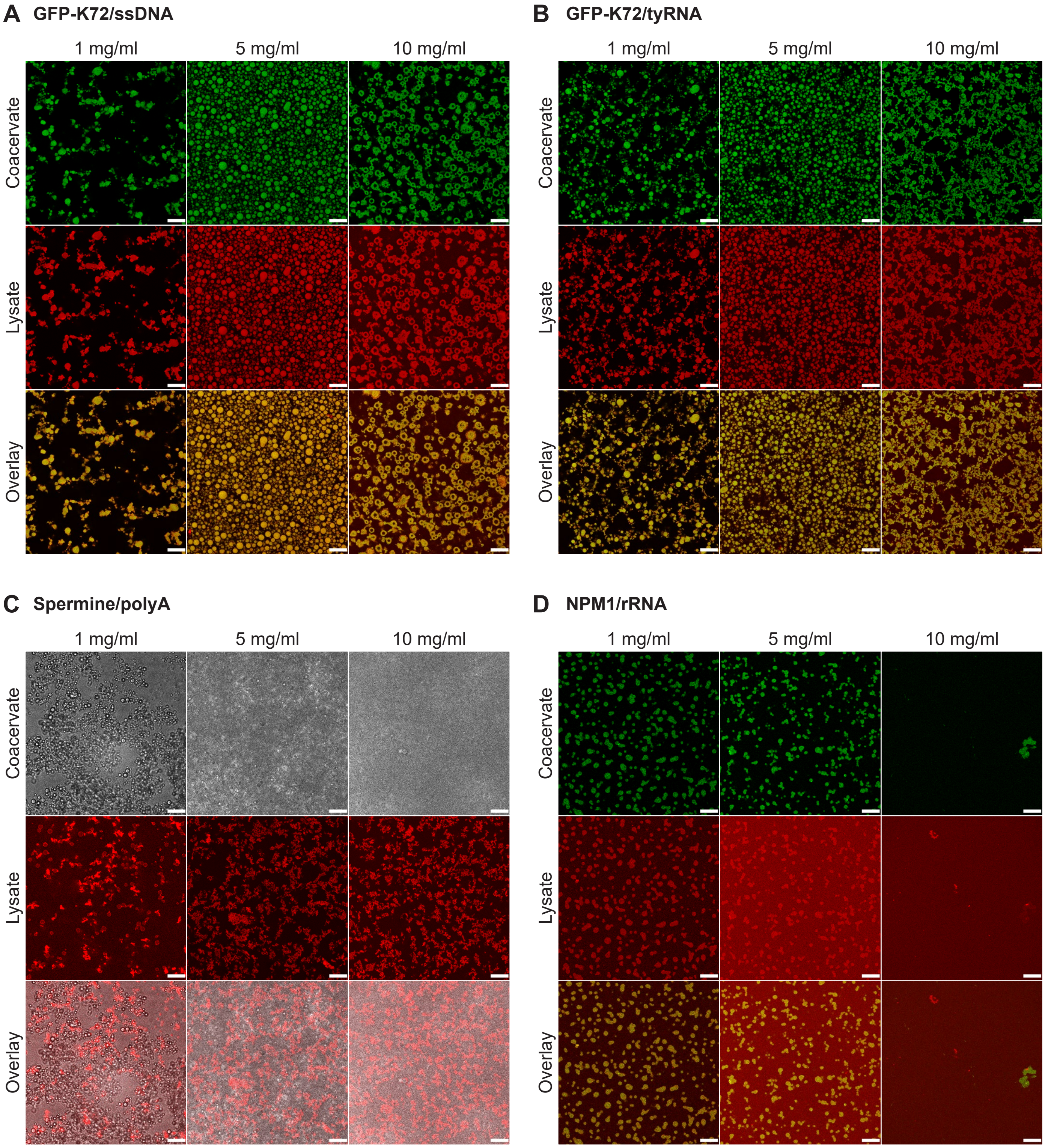
A GFP-K72/ssDNA
B GFP-K72/tyRNA
C Spermine/polyA
D NPM1/rRNA
1 mg/ml
5 mg/ml
10 mg/ml
Coacervate
Lysate
Overlay

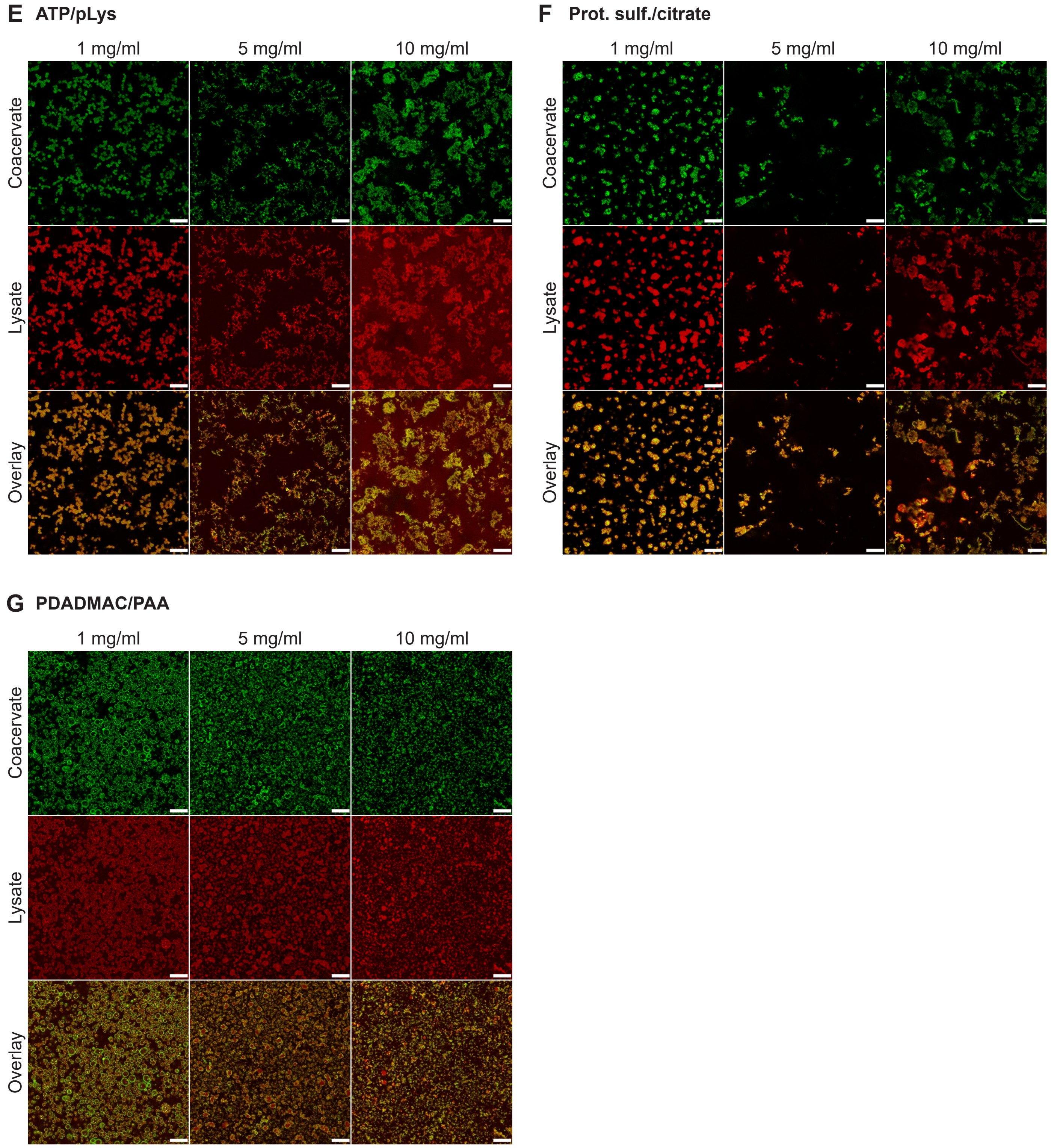

Figure S4. Bacterial cell lysate sequestering in seven systems. Following the ideal mixing condition indicated in Figure S3. Effect of increasing lyste concentration on droplet stability and morphology. Sample lysate concentration indicated as total protein concentration in mg/ml. Green channel = coacervate component/ coacervate dye. Red channel = lysate-AF647. All scale bars represent 20 µm. (A) GFP-K_{72}/ssDNA, multiphase structures can be observed at 10 mg/ml lysate. (B) GFP-K_{72}/tyRNA, some multiphase structures can be observed at 10 mg/ml lysate. (C) Spermine/polyA, aggregation can be observed. (D) NPM1/rRNA, droplet number decreased starkly at 10 mg/ml lysate, most likely due ionic strength of total mixture. (E) ATP/ pLys, aggregation can be observed. (F) Prot. sulf./citrate, aggregation can be observed. (G) PDADMAC/PAA, aggregation can be observed. Coacervate dyes (labeled components) are the same as in Figure S3, except for spermine/polyA, where no labeled component was used. Additional salts and buffers are the same as in Figure S3.

Feeding buffer						
**Component**	**Final conc. (mM)**	**Counterion conc. (mM)**	**Final conc. (mM)**	**Counterion conc. (mM)**	**Final conc. (mM)**	**Counterion conc. (mM)**
	**Standard IVTT**		**IVTT uptake**		**Low ionic strength IVTT**	
HEPES pH 8 (K)	50	~15	50	~15	50	~15
ATP (Na)	1.5	1.5	1.5	1.5	1.5	1.5
GTP (Na)	1.5	1.5	1.5	1.5	1.5	1.5
CTP (Na)	0.9	0.9	0.9	0.9	0.9	0.9
UTP (Na)	0.9	0.9	0.9	0.9	0.9	0.9
AAs (Na, Cl)	30*	1.5†	30*	1.5†	10*	0.5†
Coenzyme A	0.26	-	0.26	-	0.26	-
β-NAD (Na)	0.33	0.33	0.33	0.33	0.33	0.33
cAMP (Na)	0.75	0.75	0.75	0.75	0.75	0.75
Folic acid (Ca)	0.068	0.068	0.068	0.068	0.068	0.068
Spermidine	1	-	1	-	1	-
3-PGA (Na)	30	60	30	60	15	30
	**Final conc. (mg/ml)**	**Counterion conc. (mM)**	**Final conc. (mg/ml)**	**Counterion conc. (mM)**	**Final conc. (mg/ml)**	**Counterion conc. (mM)**
tRNA (*E. coli*)	0.2	-	0.2	-	0.2	-
**Additional components**						
**Component**	**Final conc. (mM)**	**Counterion conc. (mM)**	**Final conc. (mM)**	**Counterion conc. (mM)**	**Final conc. (mM)**	**Counterion conc. (mM)**
	**Standard IVTT reaction**		**IVTT uptake**		**Low ionic strength**	
K (glut)	80	80	40	40	40	40
Mg (glut)	10	10	3	3	3	3
Maltose	15	-	7.5	-	0	-
	**Final conc. (nM)**	**Counterion conc. (mM)**	**Final conc. (nM)**	**Counterion conc. (mM)**	**Final conc. (nM)**	**Counterion conc. (mM)**
DNA	5	-	-	-	10	-
	**Final conc. (wt%)**	**Counterion conc. (mM)**	**Final conc. (wt%)**	**Counterion conc. (mM)**	**Final conc. (wt%)**	**Counterion conc. (mM)**
PEG8000	2	-	-	-	2	-

Table S5. Final concentrations of charged species of various IVTT compositions. Combined table of regular and reduced ionic strength IVTT reaction conditions used in this work, including counterion concentrations. Standard IVTT: typical component concentration in standard IVTT reaction. IVTT uptake: used in Fig. 2B and Fig. S6 at 0.5x. Reduced ionic strength: component concentrations used in Fig. 3A and beyond, unless specified otherwise.

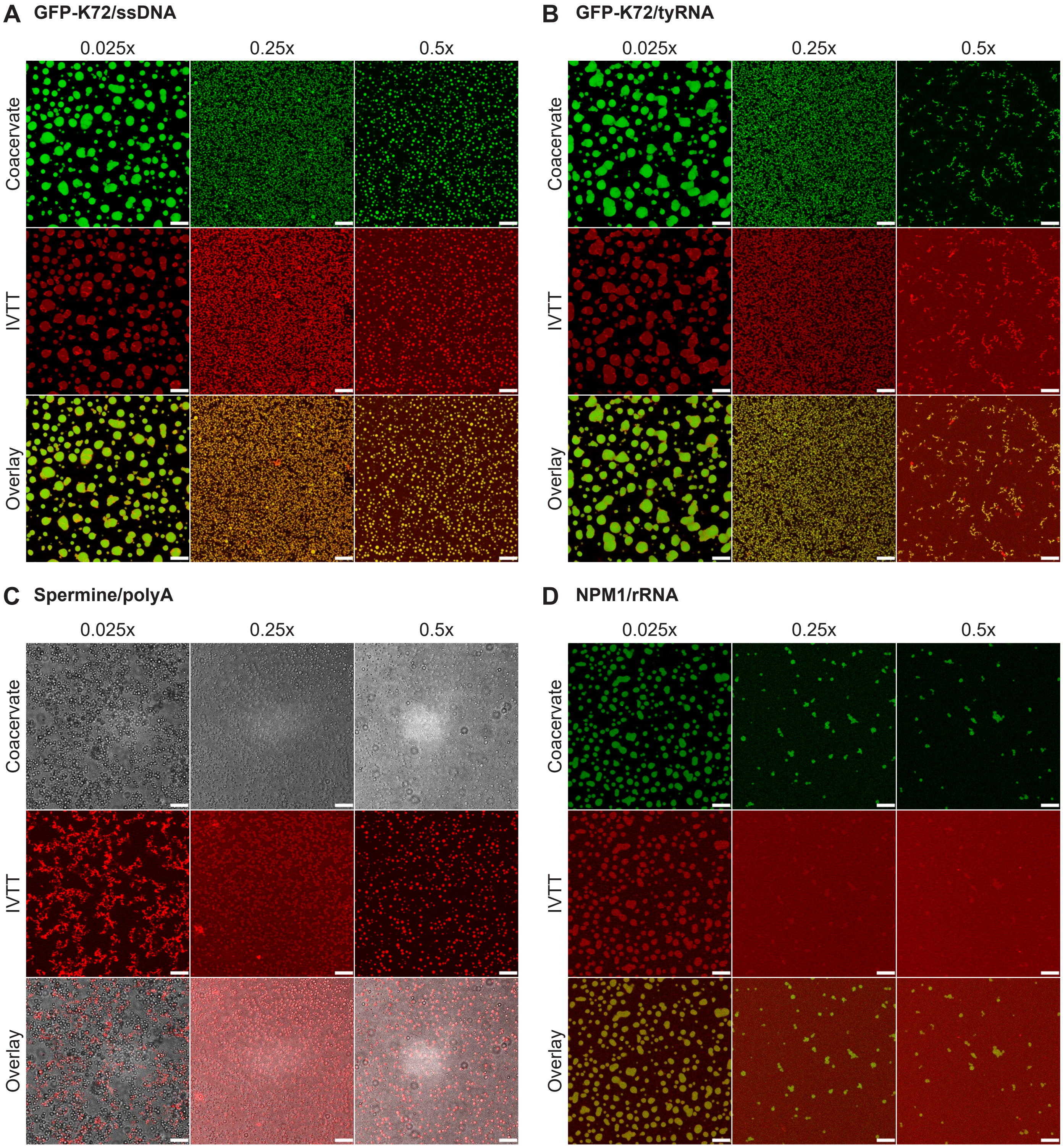
A GFP-K72/ssDNA
B GFP-K72/tyRNA
C Spermine/polyA
D NPM1/rRNA
0.025x
0.25x
0.5x
Coacervate
IVTT
Overlay

E **ATP/pLys**

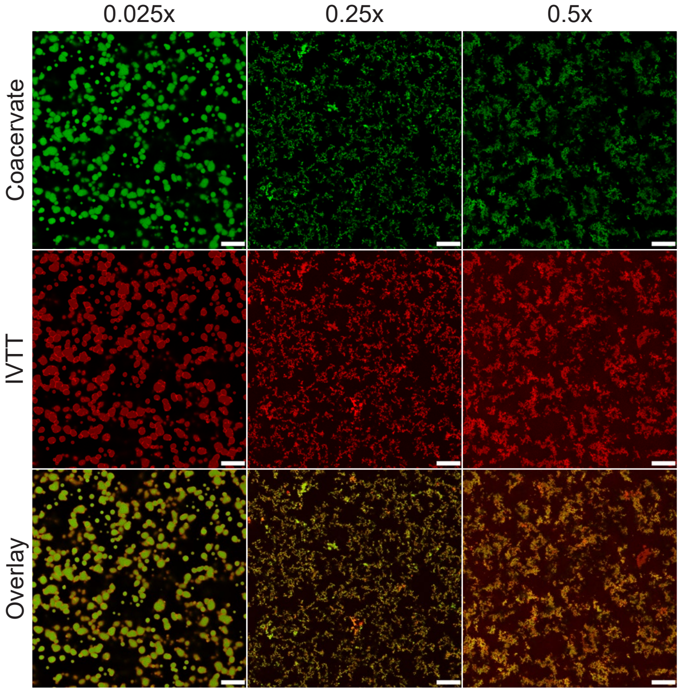

F **Prot. sulf./citrate**

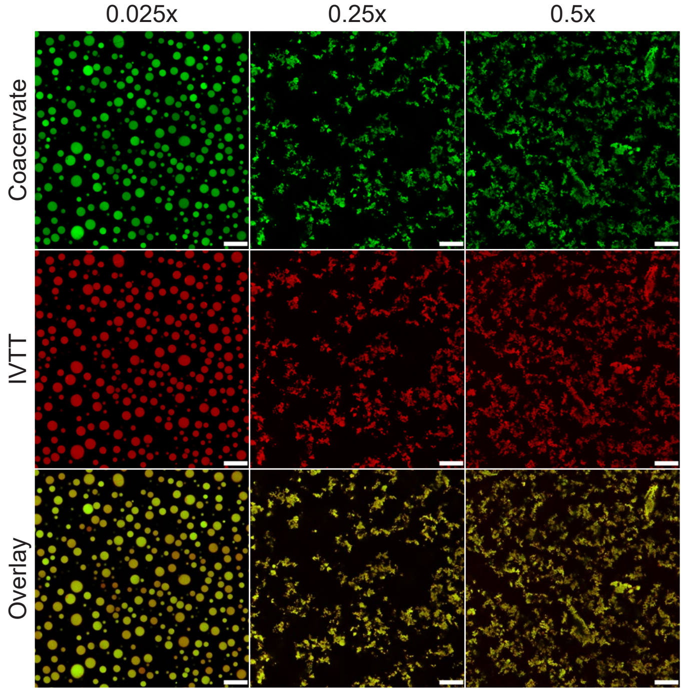

G **PDADMAC/PAA**

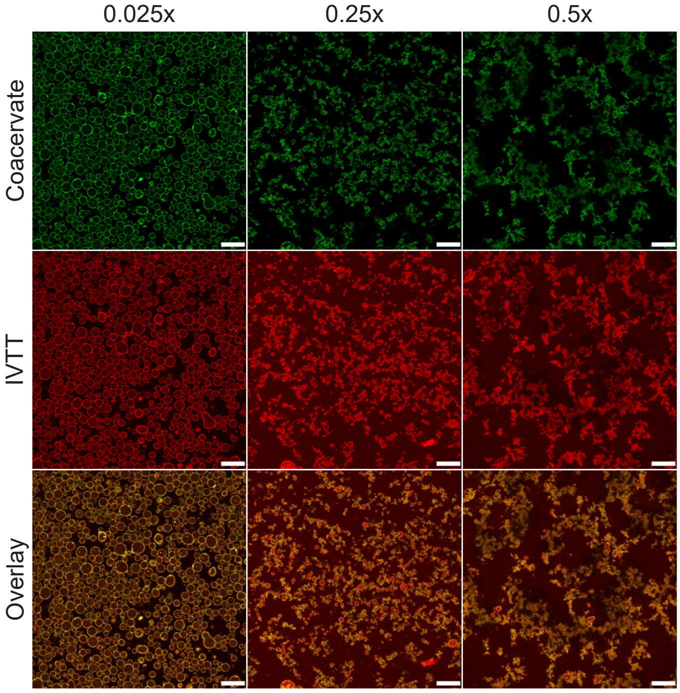

Figure S6. IVTT sequestering in seven systems. Exploring the effect of an IVTT mixture (lysate plus feeding buffer) on droplet stability and morphology. Addition of an IVTT reaction mixture (no DNA) to seven systems up to 0.5x the standard IVTT fraction. Green channel = coacervate component/coacervate dye. Red channel = lysate-AF647. Scale bars 20 µm. (A) GFP-K_{72}/ssDNA, compared to Fig. S4A, multiphase structures can no longer be observed. (B) GFP-K_{72}/tyRNA, seems less stable than the ssDNA variant. (C) Spermine/polyA, is stabilized by feeding buffer components. (D) NPM1/rRNA, ionic strength influences droplet number. (E) ATP/pLys, aggregation can be observed. (F) Prot. sulf./citrate, aggregation can be observed. (G) PDADMAC/PAA, aggregation can be observed. Coacervate dyes (labeled components) are the same as in Figure S3, except for spermine/polyA, where no labeled component was used. Additional salts and buffers are the same as in Figure S3, except for NPM1/rRNA, where 150 mM NaCl has been left out.

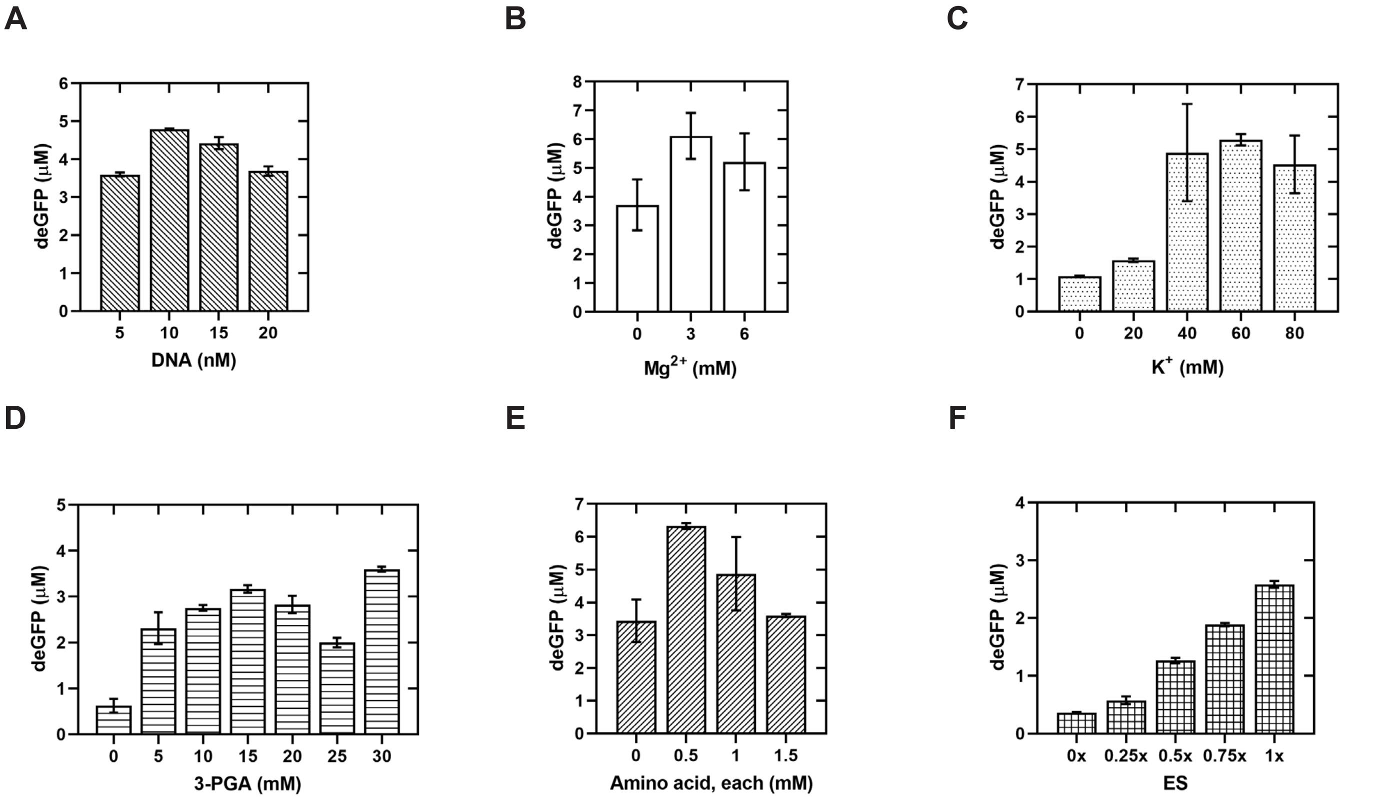

Figure S7. Expression of deGFP as a function of IVTT composition. Reducing concentrations of various feeding buffer components, and Mg/K-glut determined the minimal ionic strength condition that still gave appreciable expression. For selected concentrations see Table S5. Except for S7A, the final p70a-deGFP linear fragment concentration was 5 nM. (A) DNA range. (B) Magnesium glutamate range. (C) Potassium glutamate range. (D) 3-phosphoglyceric acid range. (E) Amino acid range, concentration per amino acid, 20 standard amino acids. (F) Energy solution dilution range. Error bars represent standard error of N = 3.

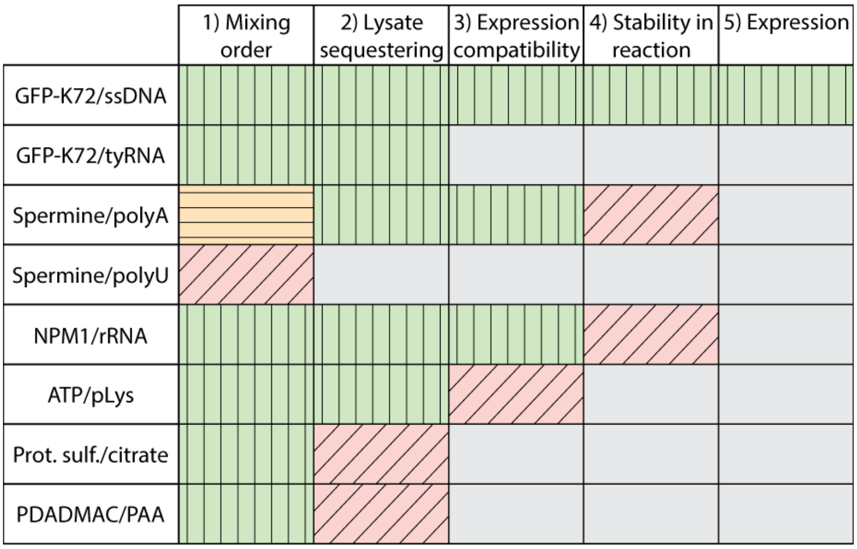

Table S8. Compatibility overview. Compatibility of each system with the corresponding step from Fig. 1. Green and vertically striped indicates broad compatibility. Red and diagonally striped indicates poor compatibility. Orange and horizontally striped indicates weak compatibility. Grey and clear indicates the systems has not been tested for the corresponding step. Definitions of when a system is considered compatible can be found in the main text. Briefly, mixing order (step 1) and lysate sequestering (step 2) required round, separated droplets without lysate aggregation, expression compatibility (step 3) required activity in the combined IVTT and droplet condition, and ideally no activity in the depleted condition, stability (step 4) required droplets to remain stable in the IVTT mixture under reaction conditions at 30 °C for 6 hours or more, and expression (step 5) required deGFP expression in the droplet sample. Some remarks: Spermine/polyU showed aggregation across all samples and was discontinued at the start. GFP-K72/tyRNA seemed less stable than the ssDNA variety. The ssDNA variety was preferred.

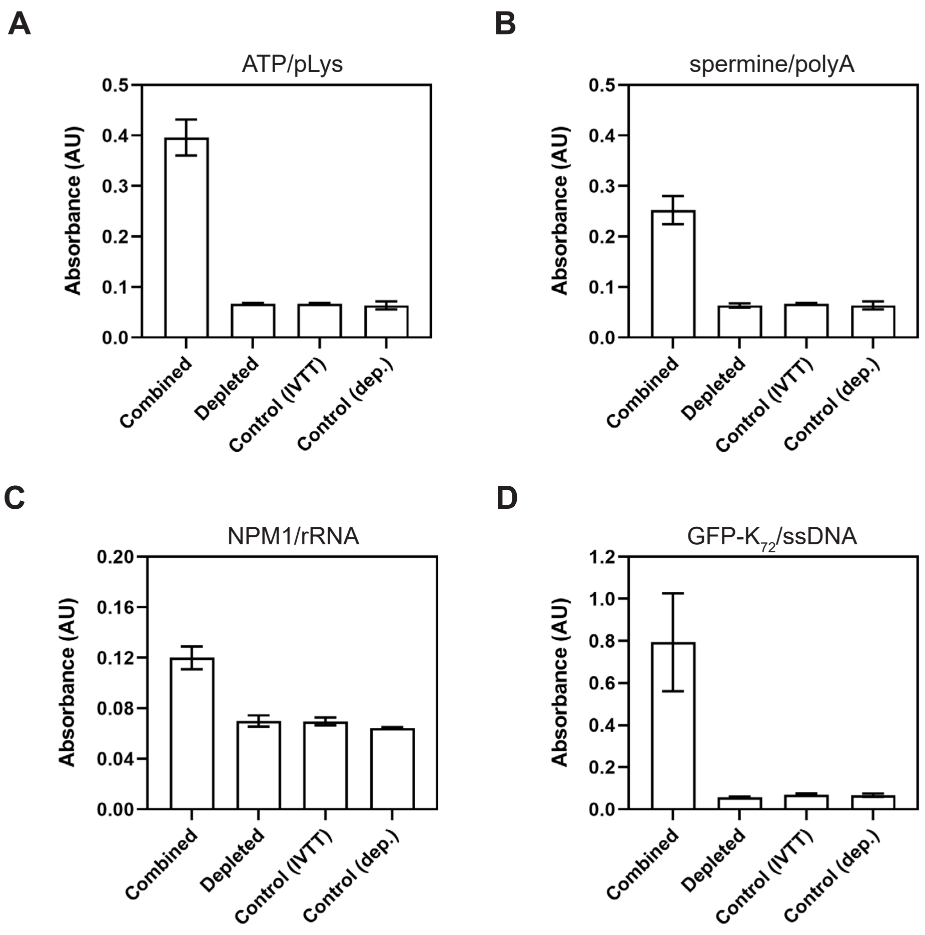

Figure S9. Turbidity measurements before and after droplet removal in four systems. Turbidity measurements at 400 nm of four systems showing that droplets were removed entirely by centrifugation. Conditions: combined = IVTT and droplets; depleted = dilute phase obtained after droplet removed by centrifugation; control (IVTT) = regular IVTT mixture before centrifugation; control (dep.) = regular IVTT mixture after centrifugation. (A) 5 mM ATP, 5 mM pLys, 5 mM Tris-HCl pH 7.4. (B) 10 mM spermine, 1 mg/ml polyA, 5 mM Tris-HCl pH 7.4. (C) 80 µM NPM1, 0.8 mg/ml rRNA, 5 mM Tris-HCl pH 7.4. (D) 12 µM GFP-K_{72}, 0.025 mg/ml ssDNA, 5 mM Tris-HCl pH 7.4. IVTT mixture consisted of 10 mg/ml unlabeled lysate and the minimal ionic strenght feeding buffer as described in Table S5, but no linear fragment. Error bars represent standard error of N = 3.

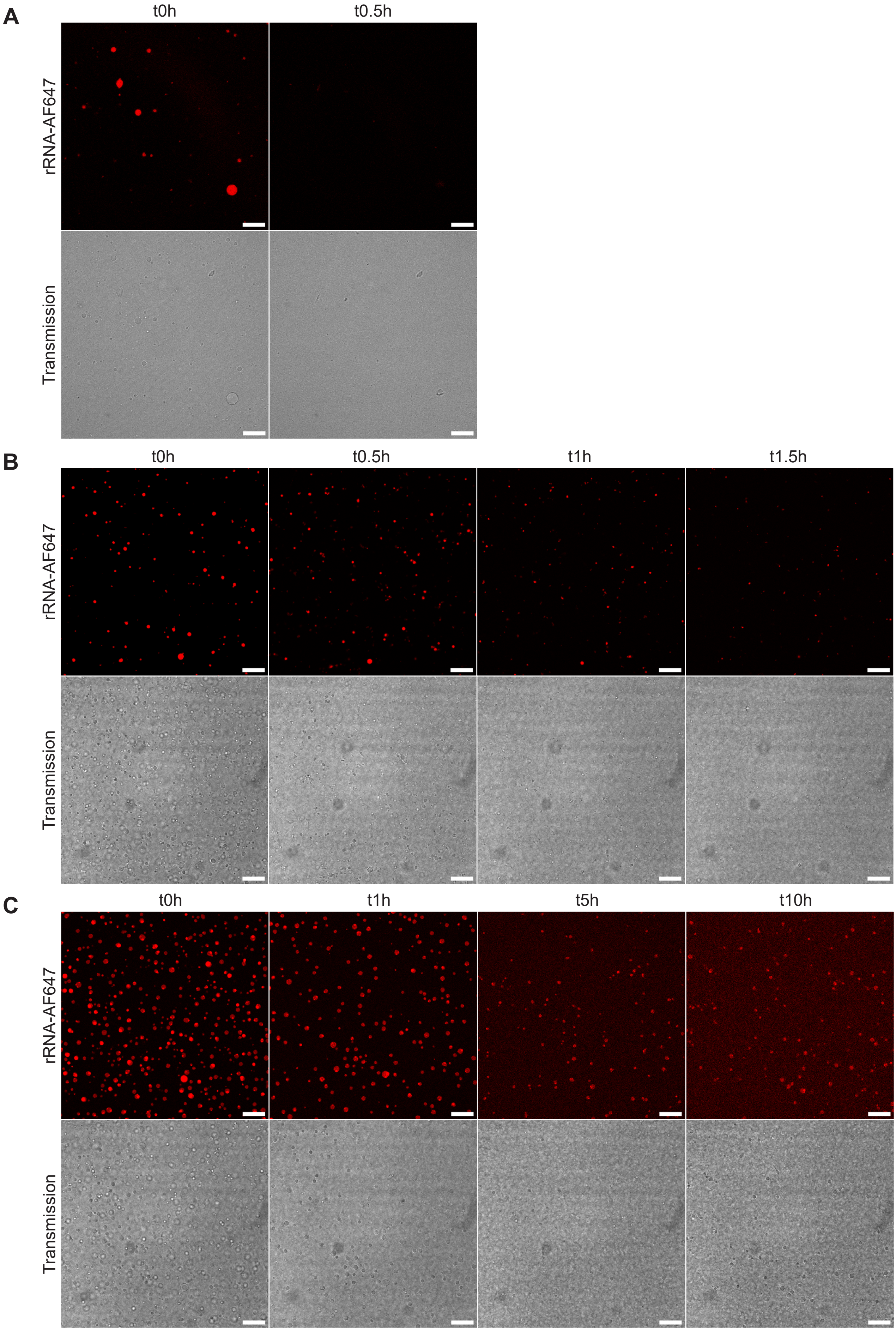

Figure S10. NPM1/rRNA droplet stability under reaction conditions. (A). 80 µM NPM1, 0.2 mg/ml rRNA-AF647, 5 mM Tris-HCl pH 7.4 droplets at 30 °C in minimal ionic strenght IVTT mixture (Table S5) with added 1 U inorganic pyrophosphatase (IPP), but no RNAse inhibitor. (B) Repeat of A with additional 1 U Ribolock at 30 °C. (C) Repeat of A with additional 1 U Ribolock at 18 °C. All scale bars represent 20 µm.

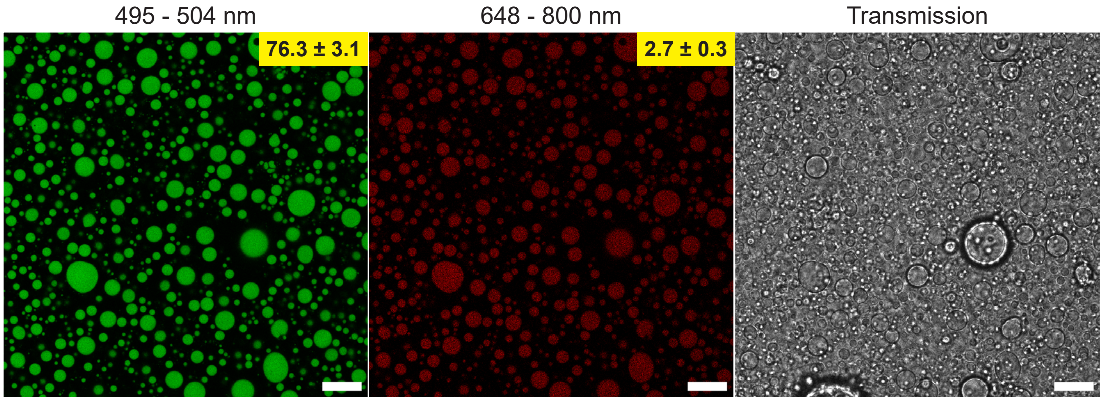

Figure S11. Bleed-through from GFP-K_{72} to mmCherry channel. Bleed-through at low laser power (0.1% 488 nm, on SP8 liachroic). Bit depth = 8. Constrast and brightness for 495-504 nm not enhanced. Contrast and brightness enhanced for 648-800 nm. Numbers indicate average pixel intensity of 5 droplets plus standard deviation. Scale bars 20 µm.

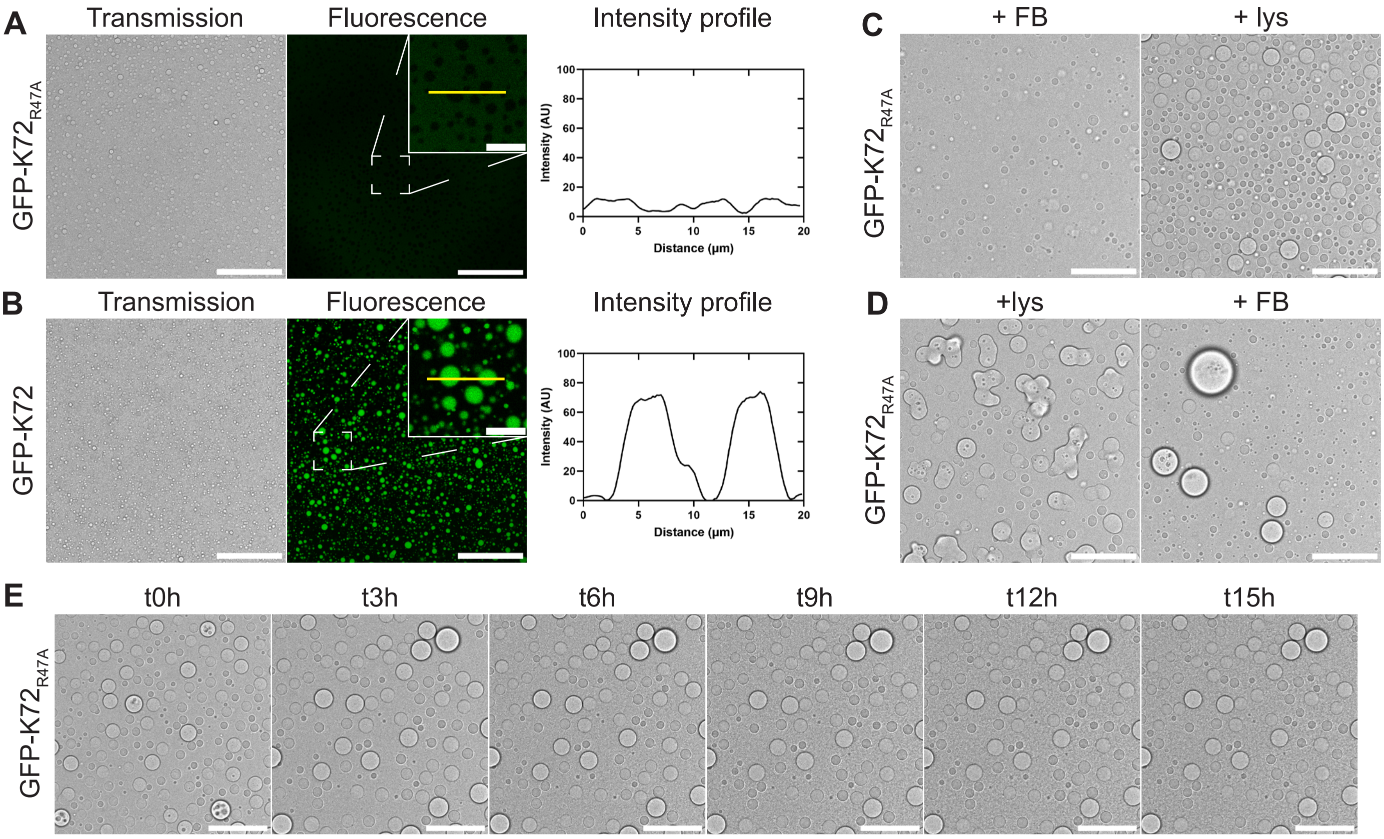

Figure S12. Phase separation and fluorescence of GFP-K_{72}-R97A mutant. (A) Fluorescence of GFP-K_{72}-R97A/ssDNA droplets at excitation 488 nm, including an intensity profile. Composition: 24 µM GFP-K_{72}-R97A, 0.5 mg/ml ssDNA, 5 mM Tris-HCl pH 7.4. Scale bars 50 µm. Inset scale bar 10 µm. (B) Fluorescence of GFP-K_{72}/ssDNA droplets at excitation 488 nm, including an intensity profile. Composition: 24 µM GFP-K_{72}, 0.5 mg/ml ssDNA, 5 mM Tris-HCl pH 7.4. Scale bars 50 µm. Inset scale bar 10. (C) GFP-K_{72}-R97A/ssDNA droplet morphology when the reduced ionic strength feeding buffer (Table S5) is added before 10 mg/ml lysate. Condensate composition same as for S12A. Scale bars 50 µm. (D) GFP-K_{72}-R97A/ssDNA droplet morphology when 10 mg/ml lysate is added before the feeding buffer mixture. Condensate composition same as for S12A. Compared to regular GFP-K_{72}-R97A/ssDNA droplets in S12A, both S12C and S12D show clear lysate uptake in terms of droplet size. Scale bars 50 µm. (E) GFP-K_{72}-R97A/ssDNA droplet stability in minimal ionic strenght IVTT (lysate, feeding buffer, no DNA) under expression conditions at 30 °C. Condensate composition same as for S12A. Scale bars 50 µm.

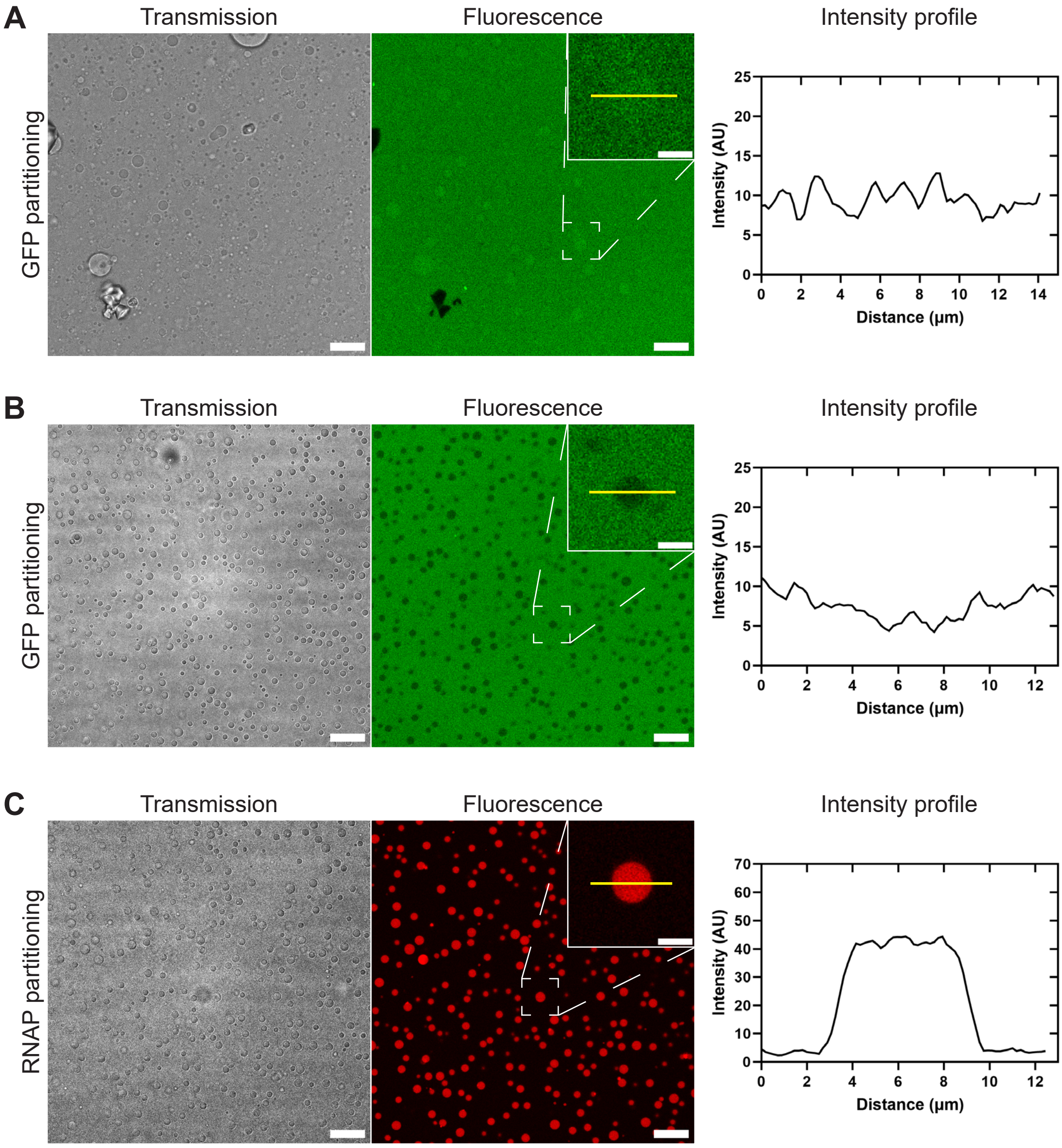

Figure S13. Partitioning into GFP-K_{72}-R97A/ssDNA droplets. (A) Partition of 7.5 µM eGFP into GFP-K_{72}-R97A/ssDNA condensates after 16 hours tube incubation in IVTT reaction mixture (10 mg/ml lysate, minimal ionic strength feeding buffer, no DNA). Composition: 24 µM GFP-K_{72}-R97A, 0.5 mg/ml ssDNA, 5 mM Tris-HCl pH 7.4. (B) Partitioning of 7.5 µM eGFP into pure GFP-K_{72}-R97A/ssDNA droplets (no IVTT) after 30 minutes incubation. Condensate composition same as in S13A. (C) Partitioning of 0.25 µM RNAP-Cy5 into pure GFP-K_{72}-R97A/ssDNA droplets (no IVTT) after 30 minutes incubation. Condensate composition same as in S13A. Scale bars 20 µm. Insert scale bars 5 µm.

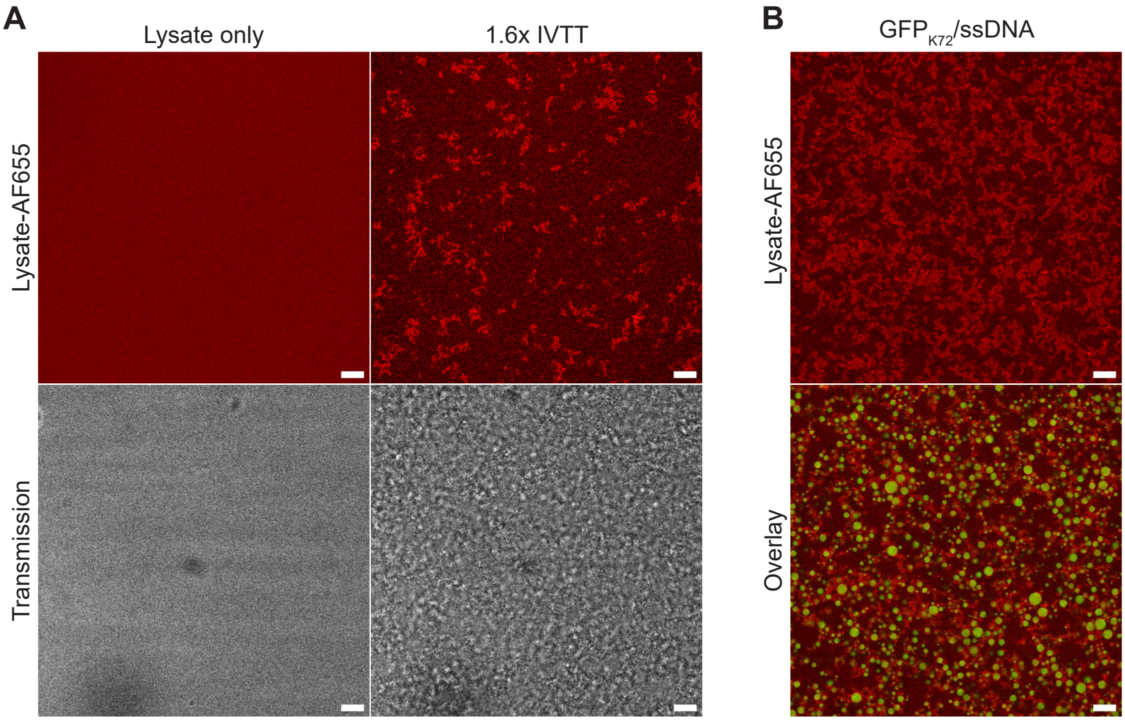

Figure S14. Aggregation of lysate and feeding buffer at high concentrations. (A) Aggregation in a 1.6x lysate and feeding buffer concentration of IVTT. (B) Addition of 1.6x feeding buffer to 16 mg/ml lysate-AF647 led to aggregation of lysate, although GFP-K_{72}/ssDNA droplets remained stable. All scale bars 20 µm.

Name	Sequence	Description
SDM-Fwd	ggttatgtTcaggaaGCGactatatctttc	Site-directed mutagenesis forward primer
SDM-Rev	gaaagatatagtCGCttcctgAacataacc	Site-directed mutagenesis reverse primer
p70a-deGFP-Fwd	ataggggttccgcgcac	For linear fragment from pTXTL-p70a-deGFP, forward primer
p70a-deGFP-Rev	ggggcggagcctatgga	For linear fragment from pTXTL-p70a-deGFP, reverse primer
R97A amino acid sequence	MASKGEELFTGVVPILVELDGDVNGHK FSVSGEGEGDATYGKLTLKFICTTGKLP VPWPTLVTTLTYGVQCFSRYPDHMKRH DFFKSAMPEGYVQEATISFKDDGNYKT RAEVKFEGDTLVNRIELKGIDFKEDGNI LGHKLEYNYNSHNVYITADKQKNGIKA NFKIRHNIEDGSVQLADHYQQNTPIGDG PVLLPDNHYLSTQSALSKDPNEKRDHM VLLEFVTAAGITHGMDELGVVGLVPRG SHMVPGVGVPGKGVPGKGVPGKGVPG KGVPGKGVPGKGVPGKGVPGKGVPGK GVPGVGVPGKGVPGKGVPGKGVPGKG VPGKGVPGKGVPGKGVPGKGVPGKGV PGVGVPGKGVPGKGVPGKGVPGKGVP GKGVPGKGVPGKGVPGKGVPGKGVPG VGVPGKGVPGKGVPGKGVPGKGVPGK GVPGKGVPGKGVPGKGVPGKGVPGVG VPGKGVPGKGVPGKGVPGKGVPGKGV PGKGVPGKGVPGKGVPGKGVPGVGVP GKGVPGKGVPGKGVPGKGVPGKGVPG KGVPGKGVPGKGVPGKGVPGVGVPGK GVPGKGVPGKGVPGKGVPGKGVPGKG VPGKGVPGKGVPGKGVPGVGVPGKGV PGKGVPGKGVPGKGVPGKGVPGKGVP GKGVPGKGVPGKGV PGWPHHHHHH	

Table S15. Primers and important sequence information. Pertains particularly to site-directed mutagenisis for GFP-K_{72}-R97A construction.

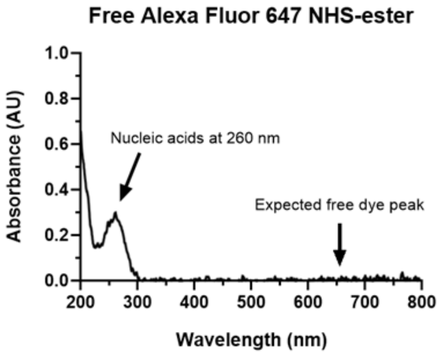

Figure S16. Spectrum of lysate-AF647 filter flow-through. Flow-through was obtained from spinning diluted lysate-AF647 through a spin filter. Spectrum shows a peak for the nucleic acids passing the filter at 260 nm, and no peak around 647 nm, indicating little to no free dye remains in the sample.
